# Allergen Delivery Inhibitors: Characterisation of Potent and Selective Inhibitors of Der p 1 and Their Attenuation of Airway Responses to House Dust Mite Allergens

**DOI:** 10.3390/ijms19103166

**Published:** 2018-10-15

**Authors:** Jihui Zhang, Jie Chen, Jie Zuo, Gary K. Newton, Mark R. Stewart, Trevor R. Perrior, David R. Garrod, Clive Robinson

**Affiliations:** 1 Institute for Infection & Immunity, St George’s, University of London, Cranmer Terrace, London SW17 0RE, UK; zhang.jihui@im.ac.cn (J.Z.); jchen99556688@outlook.com (J.C.); mrzuojie@126.com (J.Z.); 2 State Key Laboratory of Microbial Resources, Institute of Microbiology, Chinese Academy of Sciences, Beijing 100101, China; 3 BOE Technology Center, BOE Technology Group Co., Ltd., Beijing 100176, China; 4 Domainex Ltd., Chesterford Research Park, Little Chesterford, Saffron Walden, Essex CB10 1XL, UK; Gary.Newton@domainex.co.uk (G.K.N.); Mark.Stewart@domainex.co.uk (M.R.S.); Trevor.Perrior@domainex.co.uk (T.R.P.); 5 Faculty of Biology Medicine and Health, University of Manchester, Manchester M13 9PL, UK; d.garrod@manchester.ac.uk

**Keywords:** Der p 1, airway inflammation, eosinophil, house dust mite, allergens, allergen delivery inhibitor

## Abstract

Group 1 allergens of house dust mites (HDM) are globally significant triggers of allergic disease. They are considered as initiator allergens because their protease activity enables the development of allergy to a spectrum of unrelated allergens from various sources. This initiator-perpetuator function identifies Group 1 HDM allergens as attractive drug design targets for the first small-molecule approach directed towards a non-human, root cause trigger of allergic disease. The purpose of this study was to: (i) identify exemplar inhibitors of these allergens using Der p 1 as a design template, and (ii) characterise the pharmacological profiles of these compounds using in vitro and in vivo models relevant to allergy. Potent inhibitors representing four different chemotypes and differentiated by mechanism of action were investigated. These compounds prevented the ab initio development of allergy to the full spectrum of HDM allergens and in established allergy they inhibited the recruitment of inflammatory cells and blunted acute allergic bronchoconstriction following aerosol challenge with the full HDM allergen repertoire. Collectively, the data obtained in these experiments demonstrate that the selective pharmacological targeting of Der p 1 achieves an attractive range of benefits against exposure to all HDM allergens, consistent with the initiator-perpetuator function of this allergen.

## 1. Introduction

A compelling case demonstrates that as well as binding allergen-specific IgE, some allergens possess bioactivities which are essential to the development and maintenance of sensitisation to a wide range of unrelated allergens [1,2]. These bioactivities define the function of the allergenic protein in its host source, but upon interaction with structural elements of human mucosal surfaces they can cause disease, especially in individuals possessing an underlying genetic predisposition. Consequently, the broader ranging these bioactivities are, the greater their influence on the pathogenesis of allergic disease. Such allergens typically exert serodominance, generating high titre allergen-specific IgE, but by being permissive for the effects of unrelated allergens they also serve as wide-ranging initiator-perpetuators of allergy [1]. This ‘functionalist’ concept of a cadre of allergens that act as bioinitiators of sensitisation to other, comparatively effete, allergens is well established in experimental models that have given insights into the mechanisms of immunological and non-immunological collateral priming, adjuvancy, or bystander effects [3,4,5,6]. The functionalist view of allergens is also understood from the perspective of longitudinal epidemiological surveys of polysensitisation where a temporal progression in reactivity to different allergens is recognised, and where discrete initiator allergens are linked strongly to the development of disease [7,8,9,10].

Group 1 allergens from HDM have been most extensively investigated as initiator allergens [2,11,12]. In HDM, they function as digestive enzymes and are excreted in faecal pellets which are inhalable by humans. Impaction of inhaled HDM faecal pellets on the airway surface releases the protease allergen, which is resistant to inactivation by natural antiprotease defences of the airways, achieving a high localised concentration of the enzyme. The cysteine protease activity of these allergens increases the probability of detection by dendritic antigen presenting cells creates a signalling environment in the airways which favours the programming and then maintenance of immune responses with an allergic phenotype [1,11,13].

The detection of inhaled allergens by antigen presenting cells is facilitated by Group 1 HDM allergens because they cleave interepithelial tight junctions (TJs) by directly attacking the extracellular domains of the TJ adhesion proteins [14]. The ensuing non-selective increase in epithelial permeability enhances the probability of contact between any inhaled allergen and antigen presenting cell networks whose dendrites become increasingly exposed to the milieu of the airway lumen [12]. A further consequence is that dendritic and other cells accumulate in increasing numbers in the airway [13]. Additionally, the effects on TJs enable allergens to engage receptors of the innate immune system which ordinarily exhibit basolateral distribution. Ligation of these receptors activates signalling which upregulates the expression of inflammatory cytokines and facilitates the release of other mediators. Some pro-allergic cytokines, notably IL-13, downregulate the expression of TJ adhesion proteins thus exacerbating the cycle of allergen delivery to antigen presenting cells and innate signalling receptors [15]. Similar permeability events have been reported for the nasal epithelium and skin [16,17] and linkage to allergy is suggested by claudin-1 expression being inversely proportional to the Th2 polarisation of immune responses [18,19]. The recent discovery that Group 1 HDM allergens have prothrombinase activity provides new insight into how this polarisation to allergic responses occurs and suggests a novel mechanism which might contribute to epithelial–mesenchymal cell transition and airway remodelling driven primarily by the innate effects of certain allergens [2,12,20,21]. The combination of allergen delivery, polarisation of adaptive responses and immunodominance by Group 1 HDM allergens is consistent with them being initiators of allergic responses to themselves and to unrelated allergens through a process of collateral priming of immune responses [2,11,12,14].

The identification and characterisation of initiator allergens creates opportunities for the design of small molecule interventions designed to target the agents whose activities promote and sustain allergic disease [2,11,12,22]. A prominent appeal of such an approach is that the targeting of a non-human, root cause trigger of disease should combine an attractive safety profile with a significantly wider range of efficacy benefits than can be achieved by targeting discrete downstream effector pathways (e.g., leukotriene antagonists, cytokine-specific biologics), or by the less selective action of corticosteroids [12,22]. Attempting this type of intervention is now feasible following biological advances in understanding the molecular basis of allergenicity, and chemical advances in structure-based protease inhibitor design [2,11,12,22]. Thus, for the first time since the invention of allergen immunotherapy by Noon and Freeman in 1911, is it possible to envisage small-molecule pharmacotherapy aimed directly at the causative, non-human, aspect of allergy [23].

We have recently disclosed the design and synthesis of novel inhibitors of Group 1 HDM allergens which target the allergy initiator-sustainer mechanisms by inhibiting their proteolytic activity [2,12,22]. Conveniently, the high degree of identity or similarity in the amino acid sequences of the Group 1 allergens from dust mites of different species means that they comprise a single target for the purposes of inhibitor design [2,11,12]. As inhalant allergens with dominant roles as initiator-sustainers of allergic asthma and perennial allergic rhinitis, direct administration of the inhibitors to the airways should neutralise inhaled allergen molecules before significant interactions with the airway lining can occur. For these reasons, our inhibitor design programme sought to identify potent molecules which possessed the pharmaceutical credentials for delivery by inhalation, together with other attributes (e.g., selectivity, safety, and endurance) necessary for clinical developability. These compounds have been named ‘allergen delivery inhibitors’ (ADIs), reflecting an important facet of the bioactivity of Group 1 HDM allergens [2,11,12,22].

In the present paper, we compare the effects of exemplar inhibitors arising from this drug design programme. Additionally, we describe the properties of a range of novel reagents and analytical methods that will facilitate further investigations of the Group 1 HDM allergens using approaches which offer substantial improvements over tool compounds available hitherto.

## 2. Results

### 2.1. Degradation of ADZ 50,059 by Group 1 HDM Allergens and Its Inhibition

Initially, we investigated the cleavage of ADZ 50,059 by Der p 1 and Der f 1 to establish whether the substrate exhibited similar behaviour against Group 1 allergens from two globally-significant species of HDM. We were also interested in comparing the susceptibility of the substrate to a range of other enzymes of interest. Functional similarity of the allergens was confirmed by their comparable, low micromolar, Km values (Table 1), consistent with predictions from their aligned amino acid sequences. ADZ 50,059 was a poor substrate for the serine proteases trypsin and chymotrypsin but, as anticipated from bioinformatics and computational chemistry, a more effective substrate for off-target human cysteine peptidases related to Der p 1 and Der f 1. That cathepsin B was less favoured than cathepsins K and S was also anticipated because of the combined effects of its slightly lower degree of sequence identity with the HDM allergens (~25% for mature sequence) compared to cathepsins K or S (31–34%) and differences in the size of substrate binding pockets in these enzymes.

**Figure 1:**
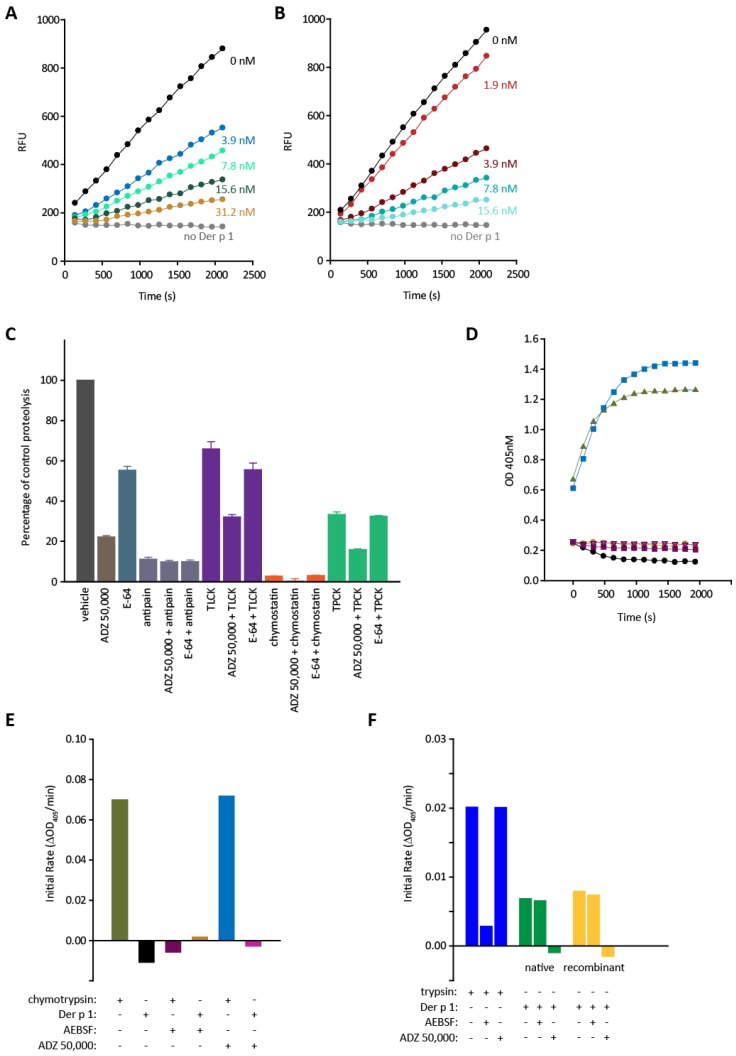
Proteolysis of ADZ 50,059 by Der p 1 or mixed HDM allergens and its inhibition. (**A**) Progress curves for cleavage by Der p 1 in the absence or presence of a range of concentrations of ADZ 50,000. (**B**) Inhibition of Der p 1 by Compound **4**. In both (**A**) and (**B**), the grey lines show data in the absence of added enzyme. Progress curves are depicted as a single data set which was replicated twice on a single 96-well plate and >3 times for each compound on separate occasions. (**C**) Proteolysis of ADZ 50,059 by mixed HDM allergens containing 40 nM Der p 1 and the effects of inhibitors tested at the following concentrations: ADZ 50,000 (50 µM); E-64 (200 µM); antipain (200 µM); TLCK (1 mM); chymostatin (50 µM); TPCK (50 µM). Data are mean ± s.e. (n = 4). (**D**) Example progress curves for the cleavage of *N*-succinyl-Ala-Ala-Pro-Phe-*p*-nitroanilide by chymotrypsin (green triangles) and its modification by 400 µM AEBSF (purple squares) or 40 µM ADZ 50,000 (blue squares). Corresponding data are presented for native Der p 1 (black circles) with AEBSF (orange circles) or ADZ 50,000 (magenta triangles) showing that this substrate is insensitive to pure Der p 1. (**E**) Example initial rates of reaction as determined from an experiment similar to that depicted in (**D**) confirming the resistance to degradation of the chymotrypsin substrate and the lack of inhibitory activity of ADZ 50,000 on chymotrypsin. (**F**) Processing of *N*-Bz-Phe-Val-Arg-*p*-nitroanilide by trypsin (blue), native Der p 1 (green), and recombinant Der p 1 (yellow) and the effects of 80 µM AEBSF or 8 µM ADZ 50,000.

Figure 1A,B shows that the rate of degradation of ADZ 50,059 by Der p 1 was retarded in the presence of ADZ 50,000 or Compound **3**. The ability of ADZ 50,000 to inhibit Der p 1, Der f 1 and other proteases was quantified by comparison of pseudo first order rate constants ($\frac{k_{obs}}{\left[I\right]}$). As expected, ADZ 50,000 was highly reactive with Der p 1 and Der f 1, but essentially inactive against selected serine peptidases (Table 2). Against cathepsins B and S it showed limited (<10-fold) intrinsic selectivity over Der p 1, whereas against cathepsins H, K, and L the difference in reactivity was considerable (~100–10,000-fold). For comparison, the generic cysteine peptidase inhibitor E-64, which has been used by others as a Der p 1 inhibitor tool, is significantly less reactive with Der p 1 than ADZ 50,000 ($\frac{k_{obs}}{\left[I\right]}$ (3.4 ± 0.23) × 10^5^ M^−1^ s^−1^) and has little selectivity.

A snapshot of ADZ 50,000 compared to a range of ‘standard’ protease inhibitors is shown in Figure 1C which depicts their effects on the cleavage of substrate ADZ 50,059 by a natural mixture of HDM allergens (i.e., containing both cysteine and serine peptidase activity). Most of the proteolytic activity was inhibited by ADZ 50,000, whereas E-64 was only a moderate inhibitor. While we did not elect to ensure that either compound completely titrated the catalytic sites of Der p 1 in the mixture, their relative performance in this assay is consistent with pseudo first order kinetic rate constant measurements reported above. Antipain and chymostatin, which discriminate poorly between serine and cysteine proteases, were effective inhibitors of substrate cleavage, whereas TLCK and TPCK, which show marginally better preference but not clear selectivity for serine proteases, were less effective and were augmented by ADZ 50,000 but not E-64.

Although not optimised for selectivity, it is clear that ADZ 50,000 lacks the indiscriminate behaviour of common inhibitor tools and highlights the limitations of those compounds in the study of HDM protease allergens. As shown in Figure 1D,E it does not inhibit the initial degradation of *N*-succinyl-Ala-Ala-Pro-Phe-*p*-nitroanilide by chymotrypsin, whereas AEBSF is, as expected, a potent inhibitor. In contrast, Der p 1 is inactive against this substrate (Figure 1D,E). Figure 1F illustrates the degradation of *N*-Bz-Phe-Val-Arg-*p*-nitroanilide by trypsin which is strongly inhibited by AEBSF but not by ADZ 50,000. This substrate is also degraded by both native Der p 1 and recombinant Der p 1 in reactions which are insensitive to AEBSF but fully attenuated by ADZ 50,000. These experiments show that despite a lack of formal optimisation and having a mechanism which involves an electrophilic attack on the catalytic residue, ADZ 50,000 discriminates well between its intended Der p 1 target and serine peptidases. Furthermore, there is no serine protease activity associated with Der p 1.

### 2.2. Effects of Der p 1 and Der p 2 on Barrier Properties of Human Airway Epithelial Cells and Its Modulation by ADZ 50,000

**Figure 2:**
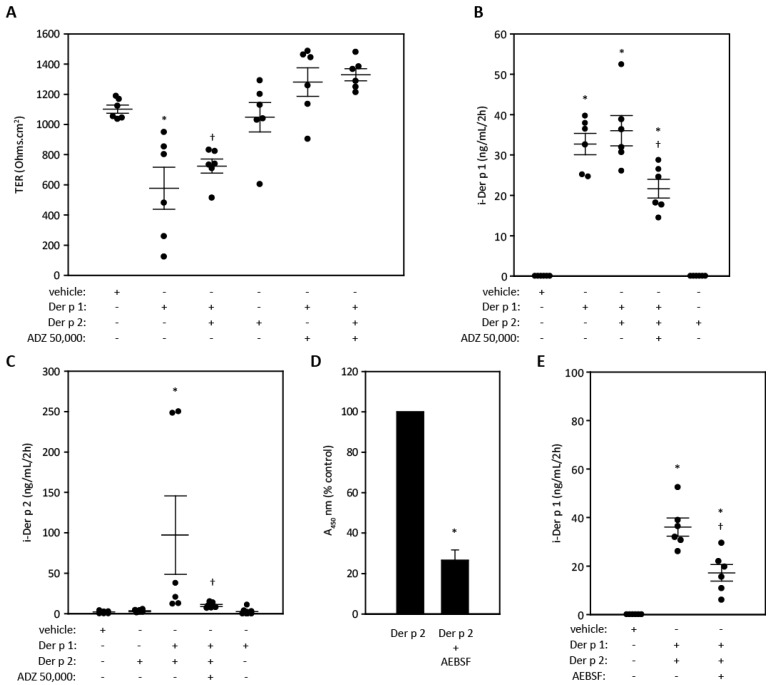
Effect of ADZ 50,000 on TER and the transepithelial disposition of Der p 1 and Der p 2 in the Calu-3 human airway epithelial cell line. (**A**) Contrasting actions of Der p 1 (2 µM) and Der p 2 (2.8 µM) on TER and the inhibition of Der p 1-mediated effects by ADZ 50,000 (1 µM). * *p* < 0.001, † *p* < 0.01 vs. vehicle control. Vehicle comprised serum-free EMEM containing 5 mM cysteine. (**B**) Immunoreactive Der p 1 (i-Der p 1) detected in the basolateral chamber of Transwells after 2 h incubation with apically-applied Der p 1, Der p 2 or Der p 1 with Der p 2 and the effect of ADZ 50,000. * *p* < 0.001 vs. vehicle control; † *p* < 0.01 vs. Der p 1 and Der p 2 in the absence of ADZ 50,000. (**C**) as (**B**), but measuring i-Der p 2, * *p* < 0.01 vs. vehicle control; † *p* < 0.01 vs. Der p 1/Der p 2 mixture. (**D**) Detection of contaminant serine peptidase activity in a commercial preparation of native Der p 2 and its inhibition by AEBSF (100 µM). Data are mean ± s.e. mean on 4 replicate tests on a single batch of allergen using *N*-Bz-Phe-Val-Arg-*p*-nitroanilide as substrate. * *p* < 0.05. (**E**) measurement of basolateral i-Der p 1 after the application of a mixture of Der p 1 and Der p 2 to the apical surface of calu-3 cells showing that AEBSF has an inhibitory effect on the serine peptidase contaminant associated with Der p 2. * *p* < 0.001 vs. vehicle control; † *p* < 0.001 vs. Der p 1 and Der p 2 in the absence of AEBSF. In (**A**–**C**) and in (**E**), data are shown as values from individual experiments with the mean ± s.e. indicated by the cross-hair and whiskers.

At confluence, Calu-3 cells developed an appreciable TER (>1000 Ohms.cm^2^) consistent with the well-established properties of these cells [24]. Application of Der p 1 to the apical side of the monolayers significantly reduced TER, whereas Der p 2 in greater molar equivalence neither had any effect itself nor altered the response to Der p 1 (Figure 2A). Pre-treatment of Der p 1 with ADZ 50,000 fully prevented the reduction in TER in monolayers treated with either Der p 1 alone or a combination of Der p 1 and Der p 2 (Figure 2A).

Addition of Der p 1 to confluent monolayers of Calu-3 cells with a well-developed TER was associated with transepithelial passage of the allergen such that it was detectable in the basolateral medium in the Transwell™ (Figure 2B). Consistent with the inability of Der p 2 to influence the TER changes evoked by Der p 1, the extent of Der p 1 transepithelial permeation was unaffected by Der p 2 (Figure 2B). However, addition of ADZ 50,000 significantly reduced the amount of Der p 1 recoverable in the basolateral medium (Figure 2B). In cells treated with Der p 2 alone, a small amount of immunoreactive material was detected in the basolateral compartment but this finding was similar to data from untreated control cells, suggesting that it may be due to cross-reactive material released from the cells (Figure 2C). Der p 2 only became detectable in the basolateral compartment in appreciable amounts when mixtures of Der p 1 and Der p 2 were added to the apical side of the chamber, and this response was significantly reduced by ADZ 50,000 (Figure 2C). These effects mirror the action of Der p 1 on TER and its own transepithelial disposition. The tendency to a larger flux of Der p 2 reflects the fact that it was present in excess over Der p 1 and that as a smaller molecule, and in the absence of other factors, its permeability would be expected to be greater than that of Der p 1.

In passing, we observed that some batches of commercially-sourced Der p 2 were associated with protease activity which could be inhibited by treatment with AEBSF (Figure 2D). As Der p 2 is not proteolytic per se, this most likely reflects the presence of contaminants with the potential to confound investigations of the bioactivity of Der p 2. An example of this is shown in Figure 2E where the transepithelial flux of Der p 1 evoked by a mixture of Der p 1 and Der p 2 was partially inhibited by AEBSF, which is not an inhibitor of Der p 1 (Figure 1F).

### 2.3. Cleavage of CLD1.1 by Der p 1

Our next aim was to study the susceptibility of the first extracellular domain of claudin 1 to cleavage by Der p 1. This domain was chosen because claudin 1 is expressed in the airways and is a useful representative of claudins with a ‘sealing type’ function [12]. Incubation of CLD1.1 with Der p 1 resulted in the generation of multiple peptide fragments which are summarised in Table 3. These sites are well conserved in the claudin repertoire of human lung. Unexpectedly, inspection of the fragments generated revealed similarities to a facile cleavage site identified by us in occludin (viz. Leu-Leu) [14,25] suggesting that it could be cleavage hot spot in both families of TJ adhesion protein. Formation of all claudin cleavage products was prevented by inactivation of Der p 1 with ADZ 50,000, but not by its treatment with AEBSF.

### 2.4. Cytokine Release from Airway Epithelial Cells and Its Modulation By ADZ 50,000

**Figure 3:**
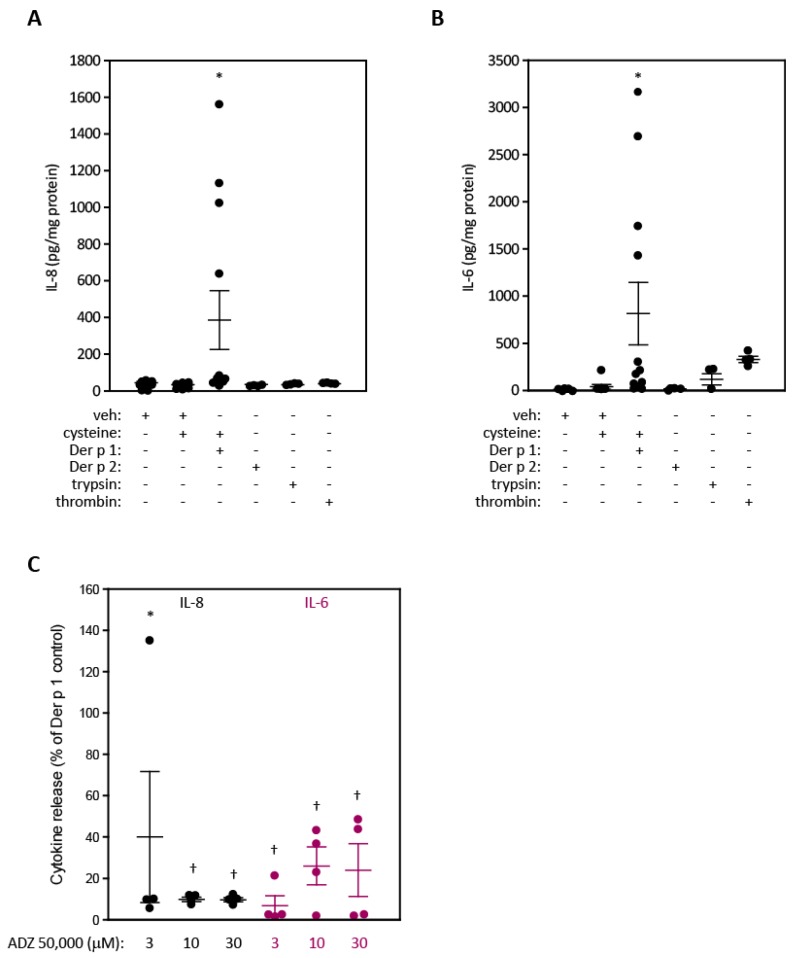
Release of IL-8 or IL-6 from cultured A549 cells and the effect of ADZ 50,000. (**A**,**B**) Release of IL-8 or IL-6 following stimulation by Der p 1 (20 nM), Der p 2 (14 nM), TPCK-treated trypsin (9 nM), or thrombin (0.5 U/mL) all applied apically. Cells were exposed for 8 h to serum-free EMEM containing the treatments, supplemented with 5 mM cysteine in the case of Der p 1. Cells were then washed and incubated with serum-free EMEM alone for a further 16 h prior to collection of samples. Data are shown as results from individual experiments with cells of different passage number. * *p* < 0.05 vs. vehicle treatments in experiments where cells were designated as responders. (**C**) Effect of ADZ 50,000 treatment on cytokine release (IL-8, black; IL-6, red) elicited by Der p 1 in designated responder experiments. Data from individual experiments are shown. * *p* < 0.05, † *p* < 0.01 vs. corresponding inhibitor-free control. In all cases, cross-hairs and whiskers denote mean ± s.e. mean, respectively.

We next investigated the ability of Der p 1 to evoke the release of IL-6 and IL-8 from airway epithelial cells and the effect of ADZ 50,000 (Figure 3A–C). The choice of the A549 cell line for these experiments was informed by literature precedent which had established them as a basis for the investigation of cytokine release by Der p 1 [26]. However, the release of both cytokines showed large variation between experiments, with some cells being refractory to activation whereas others were not. The reasons for this separation into responders and non-responders are unknown but are not related to the proteolytic competence of the Der p 1 preparations. Under identical conditions, Der p 2 was inactive in all tests and was similarly lacking in efficacy when other treatment protocols were evaluated in both A549 and calu-3 cells. Thrombin and trypsin, representative activators of PAR-1/PAR-4 and PAR-2, respectively, elicited no detectable change in IL-8 release. However, IL-6 was detectable after addition of thrombin (Figure 3A), implying the existence of PAR-1/PAR-4-dependent signal transduction in its release. Similar results to those described above were obtained when cells were incubated with these enzymes for up to 24 h and when studies were performed in calu-3 cells. Despite the variation in cytokine release overall, those experiments which were characterised by responder cells enabled us to examine whether these effects were dependent on Der p 1. Figure 3C shows the inhibitory effect of ADZ 50,000 on the release of IL-6 and IL-8 for cell passages identified as cytokine responders, consistent with the protease activity of Der p 1 in enabling their release.

### 2.5. Lung Function and Cell Recruitment Studies in Sensitized Rats

**Figure 4:**
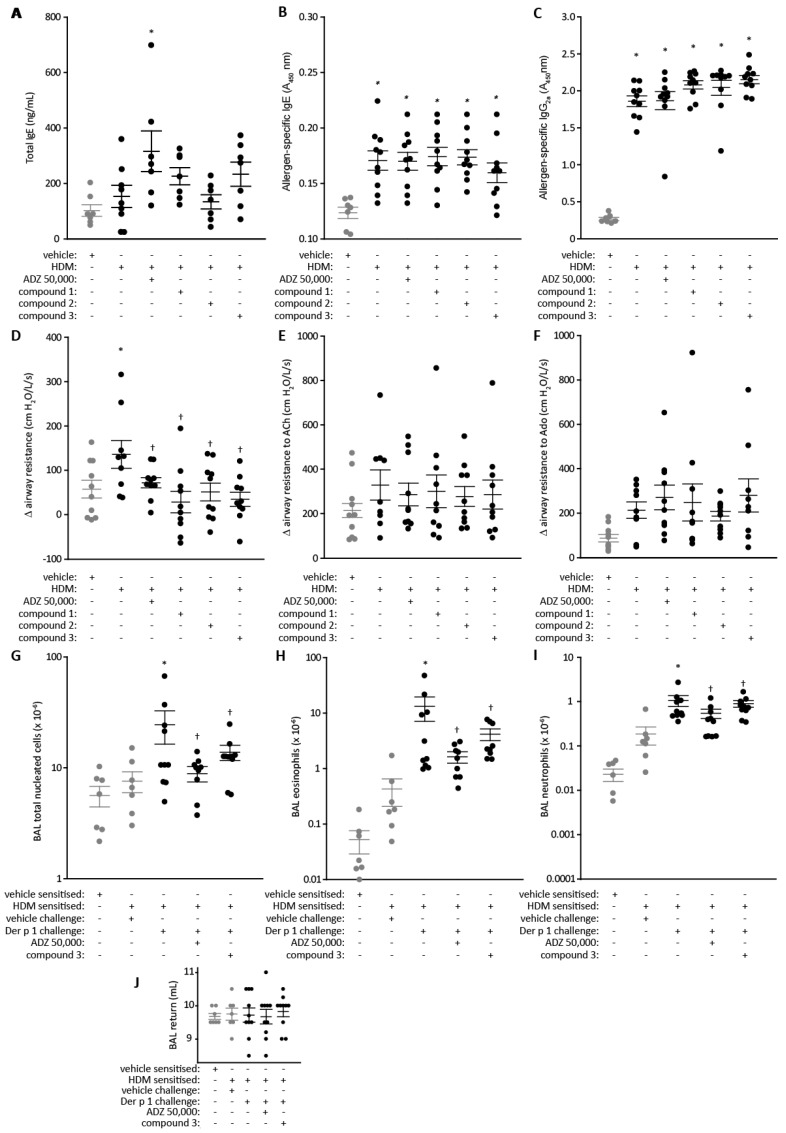
Effects of allergen delivery inhibitors on airway responses to either an extract of mixed HDM allergens or Der p 1 in brown Norway rats. (**A**–**C**) Post-sensitisation serum concentrations of IgE, allergen-specific IgE and allergen-specific IgG_2a_ in animals assigned to the treatment groups for the lung function investigations performed in (**D**–**F**). Assignment to drug treatment was made blind with respect to these data which were obtained after the conclusion of the lung function studies. * *p* < 0.001–0.05 vs. control animals assigned to sham sensitisation with vehicle (shown in grey). (**D**) Changes in airway resistance following challenge of HDM-sensitised animals with an i.t. aerosol of mixed HDM allergens (10 µg Der p 1 equivalent) or vehicle. Animals were treated with ADZ 50,000 (×10 molar ratio to Der p 1), Compound **1** (×250 molar ratio to Der p 1), Compound **2** (×200 molar ratio to Der p 1), Compound **3** (×8 molar ratio to Der p 1) prior to HDM challenge. * *p* < 0.05 vs. control animals assigned to sham sensitisation and challenge with vehicle (data shown in grey). † *p* < 0.01–0.05 vs. challenged with mixed HDM allergens. (**E**,**F**) Changes in airway resistance to acetylcholine and adenosine, respectively, measured 1 h after allergen challenge in the six treatment groups. (**G**–**J**) BAL cell profiles and BAL fluid recovery in animals sensitised to mixed HDM allergens and challenged with Der p 1 (10 µg in 100 µL) with or without prior treatment with ADZ 50,000 (×2.5 molar ratio to Der p 1) or Compound **1** (×8 molar ratio to Der p 1). * *p* < 0.01–0.05 vs. control animals assigned to sham sensitisation and challenge with vehicle or vehicle challenge in animals sensitised to mixed HDM (data shown in grey). † *p* < 0.05 vs. animals challenged with Der p 1. Data are shown as results for individual animals with mean ± s.e. indicated by cross-hair and whiskers.

In brown Norway rats, sensitisation to mixed HDM allergens produced variable effects on post-study total serum IgE compared to the vehicle-treated group (Figure 4A) but there were consistent elevations in serum allergen-specific IgE and IgG_2a_ compared to the vehicle-treated group (Figure 4B,C).

Challenge of sensitised rats with an intratracheal aerosol of mixed HDM allergens (HDM 10) evoked an acute increase in lung resistance (Figure 4D) which returned to baseline within 60 min. This effect of HDM challenge was significantly greater than the response to vehicle seen in sensitised animals. In contrast, lung resistance changes in animals challenged with HDM allergens treated with ADZ 50,000 or Compounds **1**, **2**, and **3**, were significantly reduced compared to the control challenge with HDM 10 (Figure 4D). As a further component of the study, we investigated whether challenge was associated with a change in airways reactivity to ACh or Ado. Although both stimuli suggested a tendency to increased airways reactivity 1 h after HDM challenge, these effects were not significant when compared to sensitised animals challenged with vehicle due to the high level of variability in responses. A similar pattern was evident in animals challenged simultaneously with HDM allergens and ADI compounds (Figure 4E,F). There was no change in airways reactivity to ACh or Ado in animals tested 24 h after HDM allergen challenge.

In the next study, we investigated the effect of two ADI compounds on the Der p 1-dependent recruitment of cells to the airways of rats sensitised to the full extract of HDM allergens. Our previous investigations had shown that the optimum time for monitoring the eosinophil component of this response, in which we were most interested, was 48 h following aerosol delivery of the allergen challenge. Electively, we therefore performed BAL at this time after challenge. The total number of nucleated cells in recovered BAL fluid was increased in animals challenged with Der p 1 compared to vehicle-challenged, HDM-sensitised rats, or naïve animals receiving only the BAL procedure. This response was partially attenuated in animals challenged with Der p 1 that had been treated with either ADZ 50,000 or Compound **3** (Figure 4G). A similar outcome was seen when eosinophil numbers were considered, with their recruitment significantly inhibited by the ADIs (Figure 4H). In contrast, the neutrophil recruitment response was not attenuated by ADIs (Figure 4I), but it should be noted that the cell sampling time in this protocol is not optimal for neutrophils whose airway infiltration and its resolution is more rapid than that of eosinophils. The number of macrophage/monocytes was similar regardless of the treatment group assigned. Figure 4J confirms that the recoveries of BAL fluid were comparable in the study groups.

### 2.6. Cell Recruitment Studies in Mice

**Figure 5:**
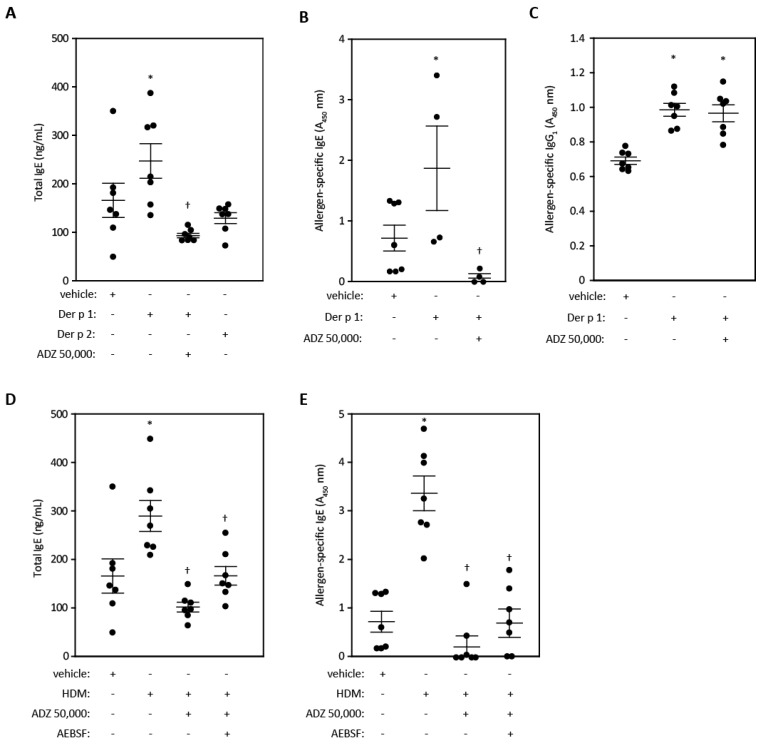
Prophylactic action of ADZ 50,000 against sensitisation to Der p 1 or an extract of mixed HDM allergens. (**A**–**C**). Serum concentrations of IgE, allergen-specific IgE and allergen-specific IgG_1_ in Balb/c mice following intranasal immunisation (10 µL/head) with vehicle, Der p 1 or Der p 2 and the effect of ADZ 50,000 on responses to Der p 1. * *p* < 0.001–0.05 vs. control animals assigned to sham sensitisation. † *p* < 0.01 vs. animals sensitised with Der p 1 (5 µg/head). (**D**,**E**) Total and HDM allergen-specific serum IgE in Balb/c mice immunised intranasally with vehicle or mixed HDM allergens (normalised to 5 µg Der p 1) and the effect of co-administered ADZ 50,000 and AEBSF formulated in a 20:1 and 25:1 molar ratio to Der p 1 content. * *p* < 0.001–0.01 vs. control animals assigned to sham sensitisation. † *p* < 0.001–0.01 vs. animals sensitised with mixed HDM allergens. In all cases, data are shown as results for individual animals with mean ± s.e. indicated by cross-hair and whiskers.

In the first of these we investigated the effect of ADZ 50,000 on the development of allergic sensitisation to Der p 1. As shown in Figure 5A, post-study levels of total serum IgE were elevated compared to the vehicle-treated mice. In contrast, animals receiving Der p 1 treated with ADZ 50,000 had serum IgE levels like the control group. A similar pattern was seen when levels of Der p 1-specific IgE were measured (Figure 5B). However, the Der p 1-dependent increase in allergen-specific IgG_1_ was not affected by pre-treatment of Der p 1 with ADZ 50,000 (Figure 5C). Unlike Der p 1, Der p 2 administered alone failed to produce an elevation in total serum IgE (Figure 5A). A further aspect of this study was to test the prophylactic effect of ADZ 50,000 on mice being sensitised to the full extract of HDM allergens. As shown in Figure 5D, the presence of ADZ 50,000 during sensitisation prevented elevation of total serum IgE and the development of allergen-specific IgE to any HDM allergen (Figure 5E). The additional presence of the serine peptidase inhibitor produced no effect beyond that of ADZ 50,000 alone (Figure 5D,E). Like the study in brown Norway rats (Figure 4), we found that the assay for allergen-specific IgE and IgG_1_ (or IgG_2a_ in the case of rats) detected a variable background in vehicle-treated animals, but in spite of this it was possible to distinguish the effect of sensitisation. We do not believe the background immunoreactivity represents prior sensitisation because the mice used in all these studies were all fully isolator-maintained and, moreover, allergen challenge did not evoke obvious allergic responses in these control animals.

**Figure 6:**
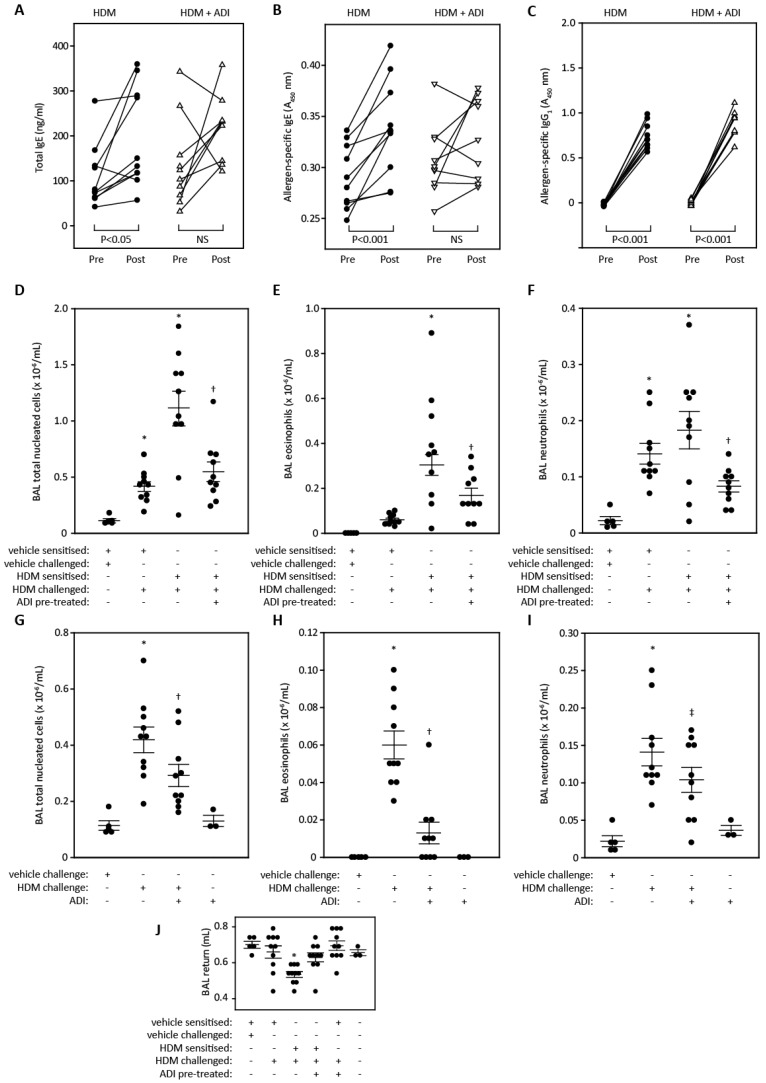
Prophylactic action of Compound **4** against i.t. aerosol sensitisation to an extract of mixed HDM allergens and its inhibition of acute allergic and innate cell recruitment in C57BL/6 mice challenged with mixed HDM allergens (25 µL, i.t., 10 µg equivalent Der p 1). (**A**–**C**) Pre- and post-immune serum concentrations of IgE, HDM allergen-specific IgE and allergen-specific IgG_1_ in mice with and without ADI treatment (Compound **4**, 10:1 molar ratio to Der p 1) during the intranasal sensitisation protocol. Responses for individual animals are shown. (**D**–**F**) BAL cell profiles in C57BL/6 mice sensitised to mixed HDM allergens following challenge with vehicle or mixed HDM allergens and the modulating effect of Compound **4**. The ADI was administered (25 µL at 40 µM, i.t.) 120 min prior to i.t. allergen provocation (25 µL, i.t.). Test compound was dosed at a 10:1 molar ratio with respect to Der p 1. The mixed HDM preparation contained a dose of 10 µg Der p 1. * *p* < 0.001–0.01 vs. vehicle challenge; † *p* < 0.01 vs. HDM challenge. (**G**–**I**) BAL leukocyte profiles in unsensitised C57BL/6 mice following i.t. challenge with vehicle or mixed HDM allergens and the modulating effect of ADI Compound **4** on the innate recruitment of leukocytes. The ADI was administered (25 µL at 40 µM, i.t.) 15 min prior to i.t. allergen provocation. * *p* < 0.001, ‡ *p* < 0.05 vs. vehicle challenge; † *p* < 0.001–0.05 vs. HDM challenge. (**J**) BAL fluid returns in the study groups. * *p* < 0.01–0.05 vs. other treatments. Data for individual animals are shown in (**D**–**J**). Cross-hairs and whiskers denote mean ± s.e. mean, respectively.

In the next study, we evaluated the effect of another ADI, Compound **4**, on the development of allergic sensitisation by comparing pre-immune and post-sensitisation IgE levels in individual animals. Figure 6A,B shows that intratracheal aerosol delivery of mixed HDM allergens in the absence of any added adjuvant increased total serum IgE and resulted in the development of allergen-specific IgE. However, inclusion of the ADI compound in the immunisation mixture prevented both responses, whereas the development of allergen-specific IgG_1_ was unaffected by ADI treatment (Figure 6C).

We then investigated the recruitment of inflammatory cells to the airways in mice sensitised to mixed HDM allergens following challenge with the same allergens. The primary purpose was to discover how these responses were affected by treatment with Compound **4** given 2 h before HDM allergen provocation. In mice challenged with HDM allergens administered by i.t. aerosol there was a significant increase in the numbers of nucleated cells recovered by BAL compared to animals which had been sham-treated with vehicle alone. This was most marked in mice actively sensitised to HDM allergens. Pre-treatment with Compound **4** prior to challenge attenuated the cell recruitment (Figure 6D). These overall effects were reflected when specific effects on eosinophils and neutrophils were considered, with the allergen-dependent recruitment of both being significantly inhibited by the single dose of Compound **4** (Figure 6E,F).

An interesting feature evident in Figure 6D–F was the ability of the mixed HDM allergens to evoke cellular responses in sham-sensitised animals that were not evident in mice challenged with the vehicle. We therefore investigated whether pre-treatment with Compound **4** influenced these innate responses also. As depicted in Figure 6G–I there were significant reductions in the overall recruitment of cells, and specifically eosinophils and neutrophils. The ADI compound itself had no effect on baseline cell numbers in BAL fluid in unchallenged animals (Figure 6G–I). Recovery of BAL fluid in the treatment groups comprising the studies depicted in Figure 6 were generally consistent except in the case of sensitised animals that were HDM challenged in whom fluid return was reduced (Figure 6J). 

**Figure 7:**
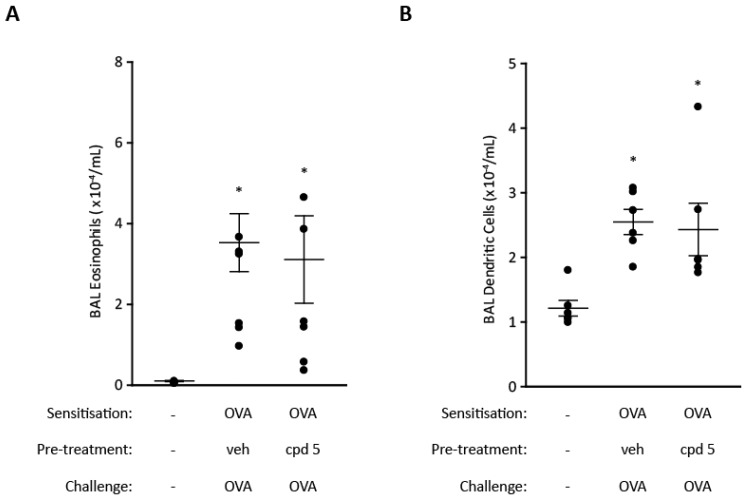
An allergen delivery inhibitor directed against Der p 1 (Compound **5**) does not affect responses to OVA in OVA-monosensitised Balb/c mice. (**A**) Recovery of CCR3^+^ eosinophils in BAL fluid of OVA-challenged animals (10 µg, i.t. aerosol) treated 2 h prior to OVA with saline vehicle or Compound **5** (50 µg/kg, i.t.) (**B**) Corresponding data for dendritic cells. Cross hairs and whiskers show mean ± s.e. mean. * *p* < 0.05 vs. control group of unsensitised animals.

To explore whether the inhibitory effects of ADI compounds on cell recruitment were due to a direct effect on Der p 1, as intended by the drug design campaign, we conducted a further study in mice sensitised to and challenged with OVA. As shown in Figure 7A,B, Compound **5** failed to attenuate the cell recruitment response following acute challenge with OVA in monosensitised mice, consistent with exemplar ADI compounds exerting their beneficial effects in HDM challenge by selective action on Der p 1.

## 3. Discussion

This paper presents novel insights into the pharmacology of protease inhibitors designed to target Group 1 allergens of HDM. We refer to this diverse class of compounds as allergen delivery inhibitors, although it will be clear from the findings presented herein that the scope of their pharmacological profile is significantly broader than implied by that name. Using exemplar ADIs from four distinct chemotypes and with differing mechanisms of inhibitory action, our studies reveal significant modulation of events ranging from innate immune responses to the recruitment of inflammatory cells to the airways following acute challenge with a mixture of HDM allergens. Unexpectedly, one feature of acute treatment with chemically diverse ADI compounds was the inhibition of acute allergic bronchoconstriction. These actions arise from a designed interaction with the Group 1 HDM allergen target and not through specific immunosuppression or bronchodilatation.

The importance of protease activity to allergenicity and the progression of allergic disease is supported by a growing range of evidence which may be briefly summarised thus: (i) facilitation of inhalant allergen detection by antigen presenting cells; (ii) activation of signal transduction mechanisms which drive innate immune responses to break tolerance and promote the acquisition of allergic sensitisation; and (iii) more chronically, activation of IgE-independent mechanisms which result in cellular transitions and airways pathophysiology of variable reversibility. In the case of (i) and (ii), it would be appropriate to consider these actions as being forms of adjuvant behaviour. These topics, specifically the target validation case for Group 1 HDM allergens, have been the subject of recent review [2,11,12].

The compounds used in these experiments are directed against Group 1 HDM allergens. As these are inhalant allergens which evoke respiratory symptoms it is therefore appropriate to consider inhalation of inhibitor drugs as a preferred route of delivery for asthma therapy. Accordingly, compounds produced in this discovery campaign were required to comply with ‘inhalation by design’ criteria during lead optimisation [2,22]. However, this is not to ignore non-respiratory effects of these allergens and compounds have been identified which may be developable for systemic use and therapeutic indications beyond asthma [22]. For asthma, because the inhibitory engagement between drug and the allergen target occurs at the luminal face of the airway epithelium, one of several factors relevant to this interaction is the composition of airway surface liquid. Airway surface liquid is rich in reducing agents, principally glutathione, which provides a means to maximise the proteolytic activity of the allergen. The pH of airway surface liquid is estimated to be in the range 6.8–7.6 [27,28,29] which is optimal for Der p 1 [30]. In severe asthma, exhaled breath condensate measurements suggest that pH may be reduced [31], but not below levels likely to materially impair enzymatic activity of Der p 1 based on assessment of its pH optimum. In fact, any such acidification would be expected to augment the protease-dependent effects of Der p 1 mediated through the generation of reactive oxygen and nitrogen species [2,32].

Using two chemically and mechanistically distinct ADIs, we showed that inhibition of Der p 1 protease activity attenuated the development of allergic sensitisation. The significance of adjuvancy provided by the protease activity of the allergen was underscored in the study using Compound **4** where mice were sensitised and challenged with the full spectrum of HDM allergens. These data clearly demonstrate that Der p 1 is an enabler/initiator of allergy to proteins of diverse structure-function. Previous studies have shown that Der p 1 facilitates sensitisation to ovalbumin and that the potentiation of allergenicity is dependent on the protease activity of Der p 1 [4,5,33]. Although capable of initiating IgE synthesis, ovalbumin differs markedly from Der p 1: The former is a comparatively effete allergen which requires an adjuvant to overcome tolerance and provide a means to cross epithelial barriers when delivered to the airways. The clear implication of our data is that protease activity of Group 1 HDM must act similarly for unrelated allergens of mite origin. Analogous findings have been made in fungi with *Aspergillus* species where protease-containing extracts of *A. fumigatus* or *A. oryzae* convert tolerogenic reactions to ovalbumin into allergic responses and, more specifically where the serine protease allergen Asp f 13 facilitates the development of allergy to the non-protease allergen Asp f 2 [3,34,35]. Given the extent and diversity of the HDM allergen repertoire, and considering that all allergens generally fall within <2% of protein families [1], it can be inferred that the adjuvant effect of Group 1 HDM allergens on the overall sensitisation to HDM allergens is a microcosm of their ability to promote allergic responses to allergens of non-HDM origin. Moreover, the significance of HDM as allergenic sources indicates that HDM protease allergens are well justified as exemplars of those allergens which enjoy exclusivity as initiators [8,10].

Whereas inhibition of the protease activity of Der p 1 attenuated the development of allergen-specific IgE and the recruitment of eosinophils following allergen challenge, it was notable that in mice neither ADZ 50,000 nor Compound **4** prevented the development of allergen-specific IgG_1_. While this may initially seem counter to the concept that Der p 1 cleaves interepithelial junctions to increase the probability of allergen detection by DCs, there are two important considerations which have a bearing on these studies. Firstly, to minimise off-target effects of non-optimised test compounds, animals were electively treated with doses incapable of completely inhibiting the amount of Der p 1 target delivered to the airways and so some immunological response could be anticipated. Secondly, the serine peptidase allergen components of the HDM allergen repertoire were not targeted and these are known to contribute to allergen delivery through an ability to cleave TJ adhesion proteins [25].

An inference from these studies is that the cysteine peptidase activity of Der p 1 is central to the initiation of allergy because its bioactivity drives both allergen delivery and the polarisation of immune responses towards IgE production. The unrestrained contribution from HDM serine peptidase allergens is, however, insufficient to drive these responses when Der p 1 is inhibited, because endogenous serine peptidase inhibitors which are known to be vulnerable to degradation by Der p 1 [36,37] provide protection against them when the activity of Der p 1 is inhibited. Other than providing a biosignature of allergen exposure, understanding the functional consequence of elevated allergen-specific IgG_1_ is complex because mouse IgG_1_ signals through both CD16 (FcγRIII), a stimulatory molecular recognition system, and CD32B (FcγRIIB), which is inhibitory, and both are widely expressed on murine haematopoietic cells [38,39]. Nevertheless, and regardless of the nuanced effects of IgG_1_, our data demonstrate that inhibition of Der p 1 evidently associates with a prophylactic reduction in the development of IgE on allergen exposure and usefully attenuates hallmark allergic effector responses when allergic sensitisation has already been established. 

Aerosol exposure of rats or mice to allergen challenge elicited an inflammatory cell response which was observed by sampling of BAL fluid. Pre-treatment with ADZ 50,000 or Compounds **3** or **4** attenuated the response to Der p 1 or mixed allergens, confirming their efficacy against the purified target and the more realistic, tougher, test posed by the allergen mixture. These experiments were undertaken with a sampling time which was optimised for the study of eosinophil recruitment, but it was pleasing to see that neutrophil recruitment was also inhibited.

The mechanism(s) by which ADIs inhibit the acute bronchoconstriction resulting from challenge with mixed HDM allergens is presently unclear. Of potential relevance is that Der p 1 degranulates human mast cells in a protease-dependent, IgE-independent manner [40]. The basis of this effect remains unknown but, interestingly, mast cells express the orphan receptor MRGPRX1 of the mas-related G-protein coupled receptor family and in airway epithelial cells this receptor has recently been suggested as a potential target of Der p 1 [41]. By analogy, the effects of the cysteine protease mucunain (the active pain-producing substance from cowhage (*Mucuna pruriens*)), is known to be a highly mast cell dependent, pseudo-allergic response [12,42]. These lines of evidence suggest that one or more mas-related G-protein coupled receptors may be the target(s) of these proteases in mast cells [12].

**Figure 8:**
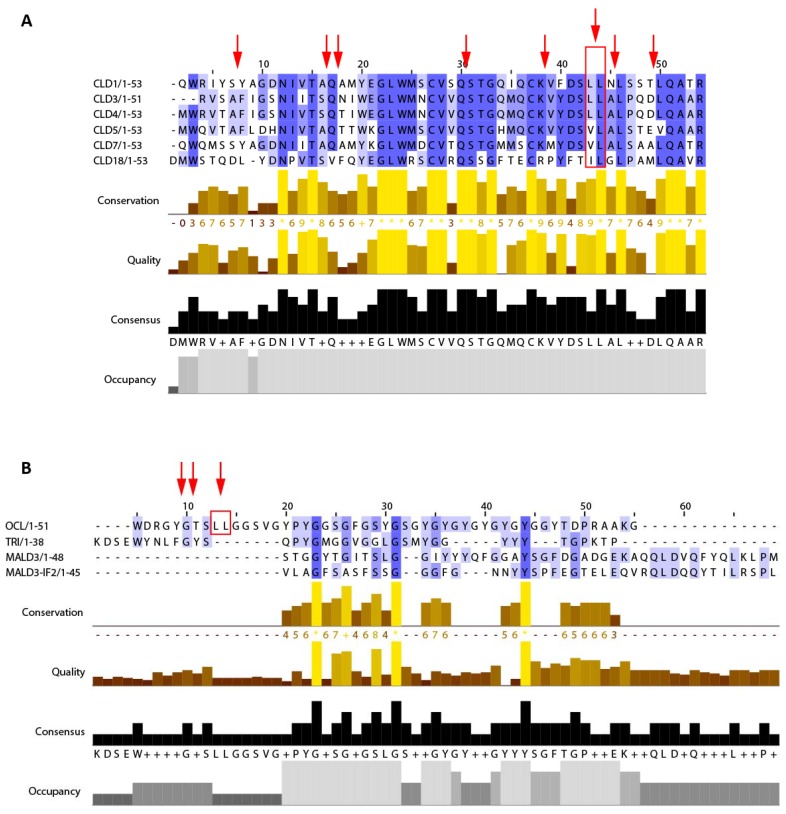
(**A**) Multiple sequence alignments of the first extracellular domain of claudins (CLD) expressed in human lung (claudins 1, 3, 4, 5, 7, and 18) and their consensus sequence created using Clustal Omega and Jalview. Colour shading denotes residues scored for conservation and quality. Arrows denote cleavage sites for Der p 1 identified in this study. (**B**) Sequence alignments of TJ-associated MARVEL domain adhesion proteins (occludin (OCL), tricellulin (TRI), MARVELD3 (MALD3)). Two isoforms of MARVELD3 are shown. Arrows indicate occludin residues cleaved by Der p 1 based on partial scanning of the first extracellular domain [14]. In both panels, the numbers appended after the protein identifier indicate the number of residues in each domain. Dyads bounded by red boxes denote putative cleavage hot spots for Der p 1 and other Group 1 HDM allergens based on the present study and previous work [14].

The prophylactic effect of protease inhibitors such as Compounds **3** and **4** in preventing sensitisation to HDM allergens raises questions about the underlying mechanism. We have previously presented evidence that small quantities of Der p 1 increase epithelial permeability to well-characterised marker solutes, suggesting that this would increase the probability of allergen detection by DCs [14,25,43,44] and showed that this effect was due, in part, to proteolysis of the extracellular domain of the TJ adhesion protein, occludin [14]. Using recombinant CLD1.1 as a model we now demonstrate that claudin family adhesion proteins possess cleavage sites for Der p 1 with similarities to principal sites identified in the MARVEL family member, occludin [14]. The choice of claudin 1 for exemplification is justified because it is present in all airway epithelial cells and archetypal of claudin family proteins which have a ‘sealing function’. Its first extracellular domain demonstrates good (40–70%) identity with the corresponding domains from most other family members (Table 4), including claudin-18, a non-classical claudin with a lung-restricted expression [15,45,46]. It shares less identity with those classical claudins (e.g., claudin 10b, claudin 15) which may be categorised as forming cation-selective components of TJs [12]. Accordingly, the Leu-Leu cleavage site for Der p 1, which is common to claudin 1 and occludin, is well represented by identity or similarity in the majority of other claudins; indeed the dyad is well conserved in the claudin repertoire (claudins 1, 3, 4, 5, 7, and 18) (Figure 7) of human bronchial and bronchiolar epithelial cells [47]. However, the cleavage sites are absent in the other TJ adhesion proteins tricellulin and MARVELD3 which are thought to have different functions from occludin and claudins (Figure 8).

Consistent with the ability of Der p 1 to attack TJ adhesion proteins we demonstrated that Der p 1 reduced TER and that this was inhibited by ADZ 50,000. This is anticipated from the prediction that claudins are cleavage targets for Der p 1 because claudins have a controlling influence on electrical resistance, whereas occludin regulates epithelial permeability to non-ionic solutes [48,49,50]. The transepithelial flux of Der p 1 was inhibited by ADZ 50,000 and this was accompanied by a reduction in the transepithelial flux of Der p 2, implying that the Group 2 allergen depended on the activity of Der p 1 to augment its transepithelial passage. In support of this, we noted that Der p 2 had no effect on TER in calu-3 cells.

A further feature arising from this programme is the characterisation of useful tool compounds for the investigation of HDM protease allergens. This is important because much previous work has relied on reagents which lack potency, selectivity, and stability. Compound ADZ 50,059 has been identified as a useful substrate for Group 1 HDM allergens offering high sensitivity and good selectivity. Illustratively, its measured K_M_ of 15.4 µM contrasts with 250–280 µM for Boc-Gln-Ala-Arg-AMC, a substrate previously used to study Der p 1 [30,36]. Compound ADZ 50,000 is a potent irreversible inhibitor of Group I HDM allergens which other investigations performed by us show to be a useful active site titrant for the quantification of functional active site concentration. To date, E-64 has been the principal inhibitor used to investigate the protease activity of Der p 1, but as shown here its broad spectrum of actions against other enzymes, including some serine peptidases, and only moderate reactivity with Der p 1 limit its utility. The novel reagents used by this study in tandem with a range of other inhibitors provides an emphatic functional rebuttal to the suggestion that Der p 1 is a multifunctional protease with both cysteine and serine peptidase activity [36,51]. A consequence of this claim has been confusion in the literature and consequent uncertainty about the meaning of data in an unknown number of studies beyond those reporting enzyme multifunctionality [36,51]. This rebuttal is the first involving direct functional data with a selective active site titrant and supports key inferences drawn when the crystal structure of Der p 1 was solved [52,53]. It seems likely that the reports of multifunctionality were consequences of using an inadequately purified protein. The easy potential for impurities in allergen preparations to lead to erroneous conclusions was highlighted in the present study where contamination of Der p 2 by proteases was shown to affect its transepithelial disposition and is reminiscent of other work in which a particularly intransigent serine protease contaminant was found associated with the major cat allergen, Fel d 1 [54]. 

In summary, the exemplar ADI compounds described in this study produce effects which suggest that an allergen-directed, inhalable small-molecule can achieve a broad range of benefits. The essential key to achieving this spectrum of desirable effects is the identification of a therapeutic target which belongs to an exclusive cadre of initiator allergens. The combination of a non-human target and an inhalable inhibitor with root cause intervention potential offers a future line of attack on the Th2 high asthma phenotype and related allergic diseases using a strategy which has a conceptually simple basis and an attractive safety profile.

## 4. Methods and Materials

### 4.1. Materials

Cell culture media and reagents were obtained from Life Technologies, Sigma-Aldrich (Poole, Dorset, UK), LGC (Teddington, Middlesex, UK), and GE Healthcare, Little Chalfont, Buckinghamshire, UK. All other materials were sourced as indicated below. Unless otherwise stated, reagents and solvents for chemical synthesis were readily available from commercial suppliers.

Analytical LC-MS was performed using a Waters ZQ instrument and purity assessed using a diode array detector. Chromatography was performed using a Phenomenex Gemini 5.0 × 3.0 mm, 5 µm, C_18_ column with a flow rate 1 mL/min using solvents A and solvent B according to the following gradient: Solvents: A: 0.1% *v/v* formic acid/water; B: 0.1% *v/v* form ic acid/MeCN; % B: ramping from 5% B to 95% B between 0 min and 3.5 min and then continuing at 95% B for 3.5–5 min.

Compounds were typically purified by standard flash column chromatography using BDH silica gel, purchased from VWR, or using a Flash Master Personal with Isolute SPE cartridges, purchased from Biotage. Where necessary, reversed phase preparative LC-MS was performed using the Waters ZQ instrument. The structures and purities of compounds were assigned using analytical LC-MS and ^1^H NMR. ^1^H NMR spectra were run on a Bruker 400 MHz instrument and spectra were analysed using MestReC. Chemical shifts are given in ppm and coupling constants (*J*) are quoted in Hz.

### 4.2. Synthesis of Der p 1 Substrate and Specific Inhibitors

(3*S*,6*S*,9*S*,12*S*,15*S*,18*S*)-1-(2-aminophenyl)-9-butyl-18-carbamoyl-15-(4-hydroxy-3-nitrobenzyl)-12-(hydroxymethyl)-3-isopropyl-6-methyl-1,4,7,10,13,16-hexaoxo-2,5,8,11,14,17-hexaazaicosan-20-oic acid (ADZ 50,059) was identified by high throughput screening (HTS) of a positional scanning library and prepared by solid-phase synthesis using conventional chemistry [55].

The following tripeptides were synthesized on Wang resin using FMoc protected amino acids and standard amide coupling procedures [55]. They were subsequently used for the synthesis of ADZ 50,000, and Compounds **1** and **2**.

**Figure:**
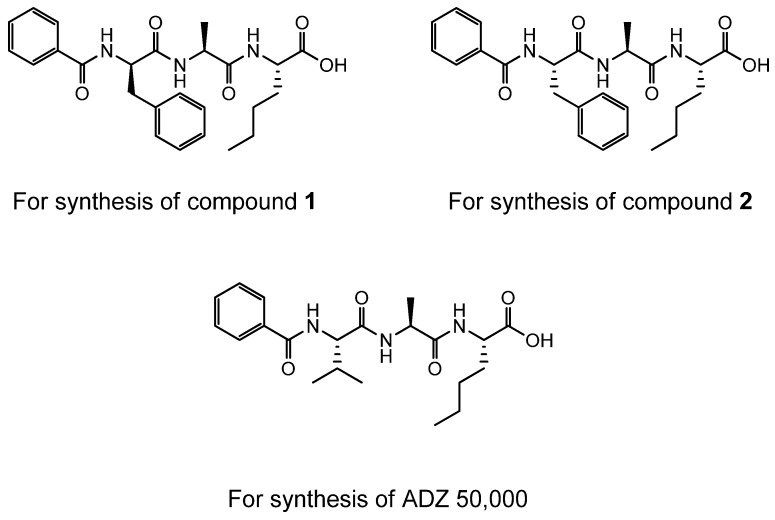


**Scheme 1:**
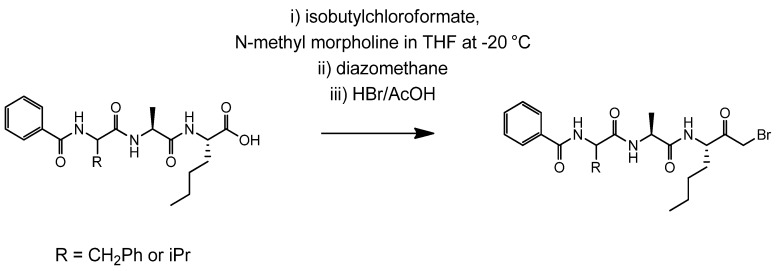
Schematic illustration.

ADZ 50,000, Compounds **1** and **2** required the synthesis of the corresponding bromomethyl ketones as described below (Scheme 1): 

To a solution of the corresponding peptide (1 equivalent) in dry tetrahydrofuran (THF) (2 mL/50 mg of peptide) and dry dimethylformamide (0.2–0.4 mL/50 mg peptide depending upon amount required to dissolve the peptide) at −20 °C (MeOH/ice) was added isobutylchloroformate (3 equivalents) and *N*-methylmorpholine (3 equivalents) and the reaction mixture stirred at −20 °C for 30 min. A solution of diazomethane in diethylether (approx. 6 equivalents; 0.15 M synthesised from Diazogen, made in accordance with Aldrich Technical Bulletin AL-180 https://www.sigmaaldrich.com/content/dam/sigma-aldrich/docs/Aldrich/Bulletin/al_techbull_al180.pdf) was added. The reaction mixture was stirred at <0 °C for 1 h then allowed to warm to room temperature and stirred for a further 1 h. A 1:1 mixture of 50% *v/v* HBr (aqueous) and acetic acid (9 equivalents, based on amount of HBr added) was then added and the reaction mixture stirred at room temperature for 30 min. Note that nitrogen is evolved, and the yellow colour disappears. The reaction mixture was subsequently diluted with ethyl acetate and washed with an equal volume of water and a saturated aqueous solution of NaHCO_3_. The organic layer was dried over MgSO_4_ and evaporated to give the desired compound as a white solid. This was used without further purification to generate ADZ 50,000, Compound **1** and Compound **2**.

**Scheme 2:**
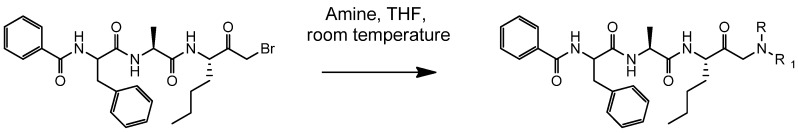
Schematic illustration.

Compounds **1** and **2** were synthesised from the corresponding bromomethyl ketone by stirring with the requisite amine according to Scheme 2.

Compound **1**, an amino ketone, (*N*-((2*R*)-1-(((2*S*)-1-((1-(dimethylamino)-2-oxoheptan-3-yl)amino)-1-oxopropan-2-yl)amino)-1-oxo-3-phenylpropan-2-yl)benzamide) was prepared via Scheme 2 as described as follows:

**Figure:**
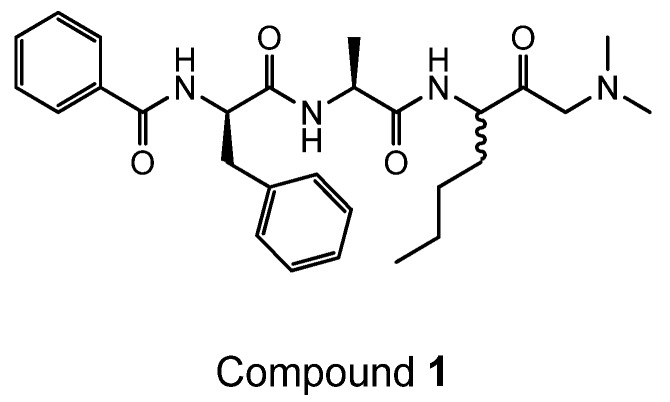


To a solution of the corresponding bromomethyl ketone (1.28 g, 1.5 mmol) in dry THF (60 mL) under nitrogen, was added a solution of dimethylamine (3.8 mL, 2M in THF) and the reaction stirred at room temperature for 6 h prior to evaporation. The crude residue was purified by reversed phased preparative LC-MS to give the title compound as a 1:1 mixture of diastereomers at the centre adjacent to the ketone, as its formate salt (269 mg, 36% over two steps); ^1^H NMR (400 MHz; *d*_6_-DMSO) δ 8.72 (1H, d, *J* = 7.3), 8.68 (1H, d, *J* = 7.6), 8.43 (1H, d, *J* = 7.3), 8.41 (1H, d, *J* = 7.3), 8.18 (2H, s), 8.02 (1H, d, *J* = 7.6), 7.99 (1H, d, *J* = 7.8), 7.84–7.78 (4H, m), 7.55–7.49 (2H, m), 7.48–7.41 (4H, m), 7.38–7.33 (4H, m), 7.30–7.24 (4H, m), 7.21–7.15 (2H, m), 4.69–4.60 (2H, m), 4.36–4.20 (4H, m), 3.45 (1H, d, *J* = 17.7), 3.30 (1H, d, *J* = 17.7), 3.22 (1H, d, *J* = 17.7), 3.15 (1H, d, *J* = 17.7), 3.09–3.01 (4H, m), 2.15 (6H, s), 2.07 (6H, s), 1.76–1.64 (2H, m), 1.64–1.41 (2H, m), 1.34–1.10 (14H, m), 0.86–0.81 (3H, m), 0.79–0.74 (3H, m); LC-MS R_t_ 1.81 min; purity 97.6%; MS *m/z* 495 (MH)^+^.

Compound **2**, also an amino ketone, (*N*-((2*S*)-1-oxo-1-(((2*S*)-1-oxo-1-((2-oxo-1-(pyrrolidin-1-yl)heptan-3-yl)amino)propan-2-yl)amino)-3-phenylpropan-2-yl)benzamide) was prepared via Scheme 2 as follows:

**Figure:**
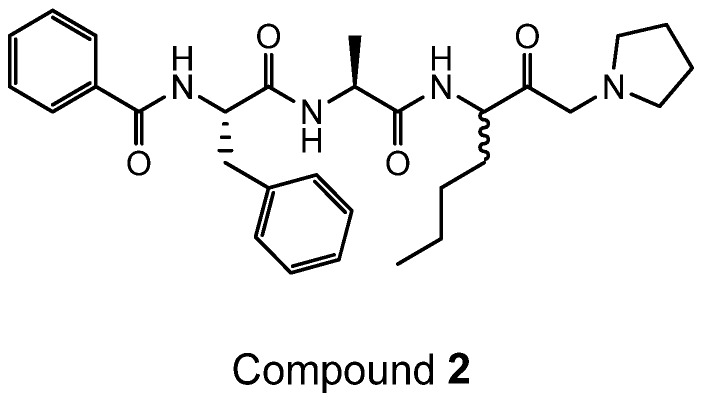


To a solution of the corresponding bromomethyl ketone (1.16 g, 2.2 mmol) in dry DCM (35 mL) was added pyrrolidine (0.40 mL, 4.8 mmol), and the reaction mixture stirred at room temperature for 4 h after which time the solvent was removed and the crude residue purified by mass-directed preparative HPLC to give the title compound as an orange solid, and a 1:1 mixture of epimers at the centre adjacent to the ketone, as a formate salt (716 mg, 58%); ^1^H NMR (400 MHz; *d*_6_-DMSO) δ 8.60 (1H, d, *J* = 8.3), 8.59 (1H, d, *J* = 8.3), 8.35 (1H, d, *J* = 7.3), 8.33 (1H, d, *J* = 7.6), 8.27 (1H, d, *J* = 7.6), 8.23–8.19 (3H, m), 7.79–7.75 (4H, m), 7.54–7.49 (2H, m), 7.47–7.41 (4H, m), 7.39–7.35 (4H, m), 7.29–7.23 (4H, m), 7.19–7.13 (2H, m), 4.76–4.67 (2H, m), 4.39–4.25 (4H, m), 3.70 (1H, d, *J* = 17.7), 3.69 (1H, d, *J* = 17.7), 3.52 (1H, d, *J* = 17.7), 3.48 (1H, d, *J* = 17.7), 3.18–3.07 (2H, m), 3.00–2.95 (2H, m), 2.66–2.55 (8H, m), 1.77–1.64 (10H, m), 1.57–1.44 (2H, m), 1.34–1.18 (14H, m), 0.90–0.80 (6H, m); LC-MS R_t_ 1.80 min; purity 98.3%; MS *m/z* 521 (MH)^+^.

**Scheme 3:**
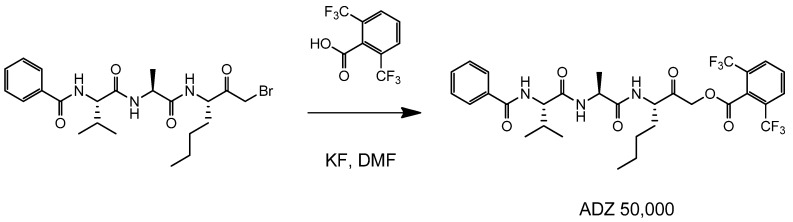
Schematic illustration.

ADZ 50,000 ((*S*)-3-((*S*)-2-((*S*)-2-benzamido-3-methylbutanamido)propanamido)-2-oxoheptyl 2,6-bis(trifluoromethyl)benzoate) was prepared according to Scheme 3:

To a solution of potassium fluoride (14 mg, 0.2 mmol, thre equivalents) and 2,6 bis-(trifluoromethyl)benzoic acid (42 mg, 0.16 mmol, two equivalents) in dry DMF (1 mL) over 4Å molecular sieve was added a solution of the bromomethyl ketone (40 mg, 0.08 mmol, one equivalent) in dry DMF (1 mL) and the reaction mixture stirred at room temperature for 3 h. The reaction mixture was then filtered through a plug of silica, washed with 10% *v/v* methanol/DCM and then evaporated to dryness. The crude residue was purified by reversed phase preparative HPLC to give ADZ 50,000 (8 mg, 15%); MS *m/z* 660 (MH)^+^.

**Scheme 4:**
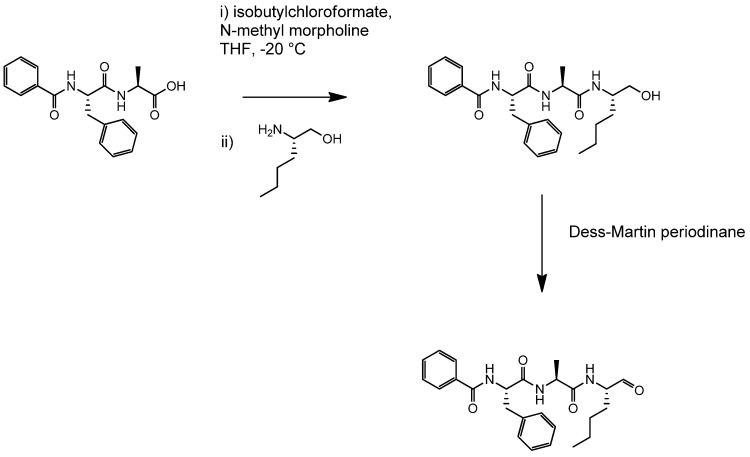
Schematic illustration.

Compound **3** was synthesised according to the following route (Scheme 4) from Bz-Phe-AlaOH which had been prepared using standard Fmoc solid phase peptide synthesis.

The intermediate *N*-((*S*)-1-(((*S*)-1-(((*S*)-1-hydroxyhexan-2-yl)amino)-1-oxopropan-2-yl)amino)-1-oxo-3-phenylpropan-2-yl)benzamide was synthesised as follows:

**Figure:**
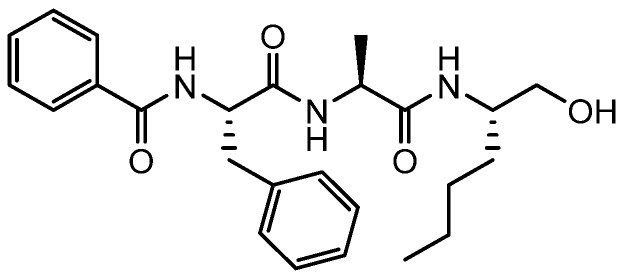


To a solution of Bz-Phe-AlaOH (374 mg, 1.1 mmol) in dry THF (12 mL) and dry DMF (0.5 mL) at −20 °C was added *N*-methylmorpholine (375 µL, 3.4 mmol) and isobutylchloroformate (157 µL, 1.2 mmol). The mixture was stirred at this temperature for 1 h then *S*-(+)-2-amino-1-hexanol (142 mg, 1.2 mmol) in dry DMF (0.5 mL) was added. The mixture was stirred at −20 °C for 2 h then allowed to gradually warm to room temperature and stirred for 18 h. The reaction mixture was subsequently diluted with ethyl acetate, washed with a saturated aqueous solution of NaHCO_3_, brine and dried over MgSO_4_. The filtrate was evaporated and the crude residue purified by flash chromatography on silica eluting with ethyl acetate to give the title compound as a white solid (360 mg, 82%); ^1^H NMR (400 MHz; *d*_6_-DMSO) δ 8.59 (1H, *J* = 8.4), 8.24 (1H, *J* = 7.3), 7.79–7.75 (2H, m), 7.54–7.48 (2H, m), 7.47–7.41 (2H, m), 7.39–7.35 (2H, m), 7.29–7.23 (2H, m), 7.19–7.13 (1H, m), 4.74–4.60 (1H, m), 4.64 (1H, t, *J* = 5.6), 4.32–4.24 (1H, m), 3.70–3.61 (1H, m), 3.36–3.29 (1H, m), 3.29–3.20 (1H, m), 3.11 (1H, dd, *J* = 13.6, 3.8), 2.98 (1H, dd, *J* = 13.6, 11.1), 1.60–1.58 (1H, m), 1.32–1.14 (8H, m), 0.86–0.79 (3H, m); LC-MS: R_t_ 2.96 min; purity > 95%; MS *m/z* 440(MH)^+^.

Compound **3**, (*N*-((*S*)-1-oxo-1-(((*S*)-1-oxo-1-(((*S*)-1-oxohexan-2-yl)amino)propan-2-yl)amino)-3-phenylpropan-2-yl)benzamide) was prepared as described below:

**Figure:**
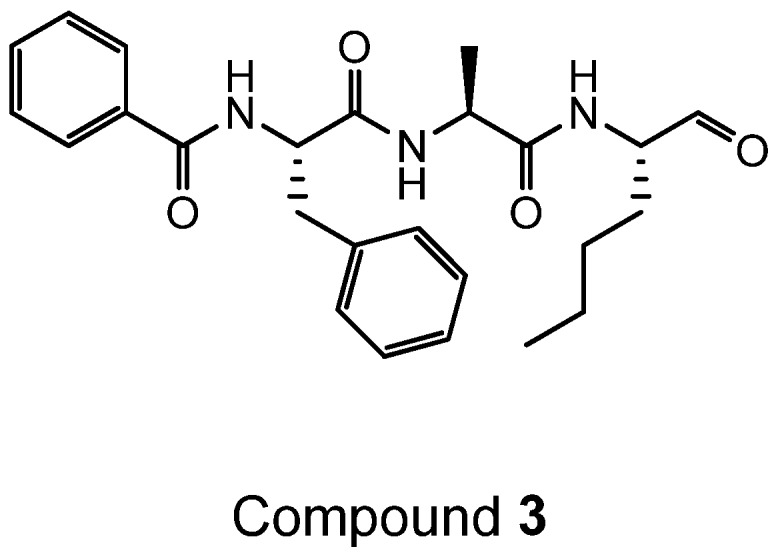


To a solution of *N*-((*S*)-1-(((*S*)-1-(((*S*)-1-hydroxyhexan-2-yl)amino)-1-oxopropan-2-yl)amino)-1-oxo-3-phenylpropan-2-yl)benzamide (180 mg, 0.32 mmol) in dry DCM (5 mL) and DMF (0.9 mL) was added a solution of Dess–Martin periodinane (15% wt solution in DCM, 1.9 mL, 0.65 mmol) dropwise and the mixture stirred at room temperature for 1 h. The reaction mixture was diluted with ethyl acetate (20 mL) and a solution of sodium thiosulphate (1 g) in a saturated aqueous solution of NaHCO_3_ (10 mL) added. The mixture was stirred at room temperature for 15 min, the organic layer separated and then washed with saturated NaHCO_3_ (aq.) (2 × 20 mL), brine (2 × 20 mL), dried over MgSO_4_ and finally evaporated to dryness. The resulting off-white solid was triturated with ether and dried under vacuum to give the title compound as a white solid (115 mg, 64%); ^1^H NMR (400 MHz; *d*_6_-DMSO) δ 9.40 (1H, s), 8.59 (1H, d, *J* = 8.1), 8.34 (1H, d, *J* = 7.3), 8.28 (1H, d, *J* = 7.1), 7.80–7.75 (2H, m), 7.54–7.49 (1H, m), 7.47–7.41 (2H, m), 7.40–7.35 (2H, m), 7.29–7.23 (2H, m), 7.19–7.13 (1H, m), 4.76–4.67 (1H, m), 4.41–4.33 (1H, m), 4.10–4.02 (1H, m), 3.17–3.10 (1H, m), 3.04–2.94 (1H, m), 1.79–1.69 (1H, m), 1.55–1.43 (1H, m), 1.34–1.20 (7H, m), 0.89–0.80 (3H, m); Purity by ^1^H NMR >95%; MS *m/z* 538 (MH)^+^.

**Scheme 5:**
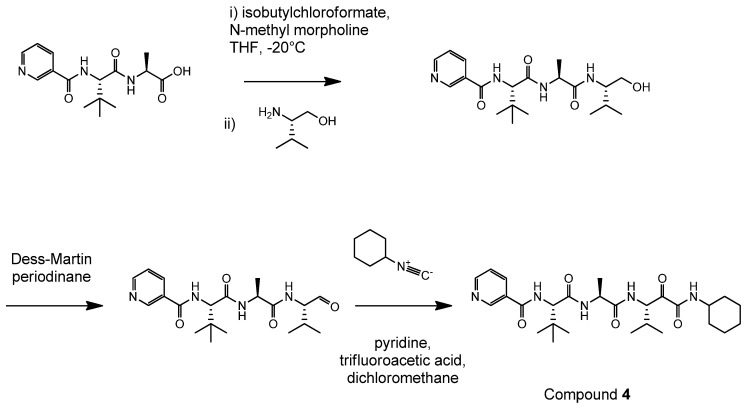
Schematic illustration.

*N*-{(*S*)-1-[(*S*)-1-((*S*)-1-cyclohexylaminooxalyl-2-methyl-propylcarbamoyl)-ethylcarbamoyl]-2,2-dimethyl-propyl}-isonicotinamide (Compound **4**) was prepared by sequential synthesis involving an aldehyde intermediate (Scheme 5). To generate this intermediate, a dipeptide acid ((*S*)-2-{(*S*)-3,3-dimethyl-2-[(pyridine-4-carbonyl)-amino]-butyrylamino}-propionic acid), was first prepared by solution-phase synthesis from an amino acid ester and Boc-(*t*-Bu)Gly-OH using methods previously described by us [22]. The dipeptide acid was then coupled with an amino alcohol in the presence of isobutyl chloroformate/*N*-methylmorpholine and oxidation with Dess–Martin periodinane performed to yield an aldehyde product. A modified Passerini reaction was then employed to produce an α-hydroxyamide from which the desired target compound, *N*-{(*S*)-1-[(*S*)-1-((*S*)-1-cyclohexylaminooxalyl-2-methyl-propylcarbamoyl)-ethylcarbamoyl]-2,2-dimethyl-propyl}-isonicotinamide, was prepared by Dess-Martin periodinane oxidation at ambient temperature until full conversion to product had occurred, as judged by LC-MS. The reaction was quenched using saturated sodium bicarbonate and sodium thiosulphate and stirred before being extracted into ethyl acetate. The extract was washed sequentially with sodium bicarbonate, deionised water, and brine before being dried and evaporated prior to purification by reversed-phase preparative HPLC. Desired product (Compound **4**) was characterised as: [M + H]^+^ 516. ^1^H NMR ((CD_3_)_2_SO, 400 MHz): δ 8.75−8.72 (2H, m, ArH), 8.57 (1H, d, *J* = 8.3 Hz, NH), 8.38 (1H, d, *J* = 9.4 Hz, NH), 8.24 (1H, d, *J* = 7.1 Hz), 8.02 (1H, d, *J* = 7.8 Hz, NH), 7.81−7.78 (2H, m, ArH), 5.04 (1H, dd, *J* = 7.8 and 5.3 Hz, CHCO), 4.52 (1H, d, *J* = 9.4 Hz, CHCO), 4.47−4.40 (1H, m, CHCO), 3.60−3.45 (1H, m, NHCH(cyclohexyl), 2.22−2.12 (1H, m, CHMe_2_), 1.72−1.64 (4H, m), 1.61−1.54 (1H, m), 1.34−1.17 (7H, m), 1.12−1.04 (1H, m), 1.00 (9H, *s,t*-Bu), 0.89 (3H, d, *J* = 6.8 Hz, CH_3_), 0.80 (3H, d, *J* = 6.8 Hz,CH_3_).

*N*-{(*S*)-1-[(*S*)-1-((*S*)-1-Benzylaminooxalyl-2-methyl-propylcarbamoyl)-ethylcarbamoyl]-2,2-dimethyl-propyl}-benzamide (Compound **5**) was synthesised as described in our previous work [22].

### 4.3. Preparation of HDM Allergen and Purification of Der p 1

Continuous solid-phase culture at 25 °C and 75% relative humidity in custom-engineered containment was used to produce populations of *Dermatophagoides pteronyssinus*. Native HDM allergen mixtures were prepared from spent culture medium according to our standard operating procedures [56]. While the conditions under which HDM are grown are known to influence the exact composition of HDM allergen mixtures, the extract produced by these procedures is designed to preserve labile bioactivity and is, to the best of our knowledge, representative of the full allergenic repertoire of HDM. The Der p 1 content of the mixtures was determined by ELISA measurement (Indoor Biotechnologies, Cardiff, UK) while proteolytic activity was measured using ADZ 50,059 as substrate. Where appropriate, ADZ 50,000 was used as a titrant to measure the concentration of functional active sites. For most in vivo studies, we used mixed HDM allergens as the reagent for sensitisation and challenge because this presentation is most representative of the material to which the airways are subjected in life. HDM mixtures were normalised by reference to Der p 1 content expressed as µg/mL as previously described [22]. According to this procedure, ‘HDM 1’ means 1 µg/mL Der p 1 and so forth. Batch-wise consistency in the activity of Der p 1 was ensured by the inclusion of a reducing agent (cysteine or dithiothreitol) in buffers or culture medium. The appropriate agent was present also in vehicle controls.

The mixed HDM allergens were also used as feedstock for the purification of Der p 1. High-purity Der p 1 was required for the characterisation of the Der p 1 substrate and for the testing of novel molecular entities designed as inhibitors of its proteolytic activity. General methods for this purification have been described in full elsewhere [56]. In brief, 2–3 volumes of Dulbecco’s PBS were added to mixed HDM allergens with overnight stirring. After centrifugation (30 min, 24,000× *g*, 4 °C), the supernatant was aspirated, and solid ammonium sulphate added to 50% saturation in the presence of 1 mM EDTA. After precipitation for >2 h the pellets were collected, and insoluble matter removed from the reconstituted solution. The soluble fraction then underwent size exclusion chromatography at pH 7.4 on an ÄktaPurifier system (HiPrep 16/60 Sephacryl S-200 HR, GE Healthcare, UK) with an elution buffer comprising 0.2 M sodium phosphate containing 0.5 M sodium chloride and 1 mM EDTA. A fraction containing Der p 1 was collected and then refined using a soybean trypsin inhibitor (SBTI) column. This cycle of size exclusion and serine peptidase removal was repeated and the final eluate passed through an Amicon ultrafiltration cell (Millipore, Bedford, MA, USA) with a 10 kDa cut-off membrane to concentrate and desalt the preparation. After dilution in 20 mM Tris-HCl buffer, pH 8.0, the sample was then chromatographed on Resource Q (GE Healthcare) using an ÄktaPurifier. Der p 1 was eluted by gradient chromatography with 0–0.5 M NaCl. Peaks containing Der p 1 were analysed by SDS-PAGE and MALDI-TOF mass spectrometry (Kratos Axima, Kratos Analytical, UK or Bruker Flex, Bruker, UK) and combined. Der p 1 was quantified by ultraviolet absorbance spectrometry in a quartz cuvette at 280 nm using an extinction coefficient of 47,705 M^−1^ cm^−1^.

### 4.4. Der p 1 and Der f 1 Enzyme Activity Assays

These were performed in 96-well plates using a PerkinElmer Multiprobe II Plus HTS EX robot (PerkinElmer, Seer Green, Buckinghamshire, UK). Reactions consisted of substrate (10 µL at 12.5 µM final concentration), reaction buffer (70 µL potassium phosphate buffer, pH 8.25 containing 1 mM EDTA) and dithiothreitol (DTT, 10 µL with a final concentration of 1 mM). Catalysis was initiated with the addition of 10 µL Der p 1 or Der f 1 dissolved in reaction buffer at 2.5 µg/mL and the reaction followed at 30 °C by measurement of fluorescence (excitation/emission 330/420 nm) using either a Fusion Alpha-FP or Envision plate reader fitted with a temperature-controlled carrier (PerkinElmer, UK). 

Using a modification of the procedure described above with mixed HDM allergens containing the equivalent of 40 nM Der p 1, studies were undertaken to compare the effects of ADZ 50,000 with a range of standard enzyme inhibitors (Sigma-Aldrich).

Enzymatic activity in a commercial Der p 2 preparation (Indoor Biotechnologies, UK) was assayed at ambient temperature using 200 µM *N*-Bz-Phe-Val-Arg-*p*-nitroanilide hydrochloride (Sigma-Aldrich, UK) and 3 µM Der p 2 in PBS containing 1 mM DTT and in the absence and presence of 100 µM 4-(2-aminoethyl) benzenesulphonyl fluoride hydrochloride (AEBSF, Sigma-Aldrich, UK). A_405_ nm was measured 15 min after reaction.

### 4.5. Assay of Cathepsin B

Assays comprised 10 µL Abz-Gly-Ile-Val-Arg-Ala-Lys-DNP-OH (Merck, Watford, Hertfordshire, UK, 5.9 µM final concentration), 70 µL of reaction buffer (0.1 M NaAc-HAc, pH 4.5, 0.2 M NaCl), and 10 µL of DTT (2.5 mM final). Reactions were initiated by adding 10 µL of human liver cathepsin B dissolved in reaction buffer to give final concentration of 0.5 nM. Reactions were performed at 30 °C and followed kinetically by excitation/emission at 320/420 nm. For inhibitor studies, the reaction buffer volume was 60 µL and inhibitor added in 10 µL aliquots.

### 4.6. Assay of Cathepsin H

Assays comprised 10 µL L-Arg-7-AMC (Sigma-Aldrich, 150 µM final concentration), 70 µL of reaction buffer (37.5 mM KH_2_PO_4_, 37.5 mM K_2_HPO_4_, 1 mM EDTA, pH 6.8), and 10 µL cysteine (3 mM final). Reactions were initiated by adding 10 µL of human liver cathepsin H dissolved in reaction buffer at a final concentration of 0.4 µg mL^−1^. Reactions were monitored at 30 °C with excitation/emission at 360/460 nm. Buffer volume was 60 µL when using an inhibitor (10 µL).

### 4.7. Assay of Cathepsin K

Assays comprised 10 µL (*Z*-Leu-Arg)_2_–Rhodamine 110 (Merck) (1.25 µM final), 70 µL of reaction buffer (50 mM MES pH 5.5, 2.5 mM EDTA, 10% *v/v* DMSO), and 10 µL DTT (2.5 mM final). Reactions were started by adding 10 µL of cathepsin K dissolved in reaction buffer to give a final concentration of 0.075 nM. Reactions were monitored at 30 °C with excitation/emission at 485/535 nm. Buffer volume was 60 µL when using an inhibitor (10 µL).

### 4.8. Assays for Cathepsin L and Cathepsin S

Assays comprised 10 µL of *Z*-Phe-Arg-AMC substrate (Sigma-Aldrich, 10 µM final concentration in the cathepsin L assay, 20 µM in the cathepsin S assay), 70 µL reaction buffer (400 mM sodium acetate buffer, pH 5.5 containing 4 mM EDTA for cathepsin L and 0.1 M sodium phosphate buffer, pH 7.5 containing 2 mM EDTA for cathepsin S), and 10 µL DTT (8 mM and 2 mM final concentrations, respectively). Reactions were initiated by adding 10 µL of human liver cathepsin L or recombinant human cathepsin S dissolved in reaction buffer to give final concentrations of 0.29 nM and 2.5 nM, respectively. Reactions were monitored at 30 °C with excitation/emission at 360/460 nm.

### 4.9. Assay of Thrombin

Assays comprised 10 µL Bz-Phe-Val-Arg-AMC (Merck, 73 µM final concentration), 70 µL of reaction buffer (10 mM HEPES, pH 8.0, 5 mM CaCl_2_, 0.02% *v/v* NaN_3_) and started by adding 10 µL of human plasma thrombin dissolved in reaction buffer (1 nM final). Reactions were performed at 30 °C and followed by excitation/emission at 370/450 nm. Inhibitor was added in 10 µL aliquots, as above.

### 4.10. Assay of Trypsin

This comprised 10 µL *N*-Bz-Phe-Val-Arg-*p*-nitroanilide hydrochloride (Sigma-Aldrich, 200 µM final concentration) and 70 µL of reaction buffer (PBS). Reactions were started at 30 °C by adding 10 µL of trypsin (2 nM final) and monitored by A_405_ nm. Inhibitor was added in 10 µL aliquots. For comparative studies using trypsin (2 µg/mL) and Der p 1 (20 µg/mL), the above conditions were modified to permit the inclusion of 10 µL DTT (1 mM final) and in these experiments AEBSF (80 µM) or ADZ 50,000 (80 µM) were used as inhibitors. 

### 4.11. Assay of Chymotrypsin

Assay mixtures comprised 10 µL *N*-succinyl-Ala-Ala-Pro-Phe *p*-nitroanilide (Sigma-Aldrich, 200 µM final), 70 µL of reaction buffer (0.1 M Tris-HCl, 10 mM CaCl_2_, 250 mM NaCl, pH 8.0). Reactions were monitored by A_405_ nm at 30 °C after adding 10 µL of chymotrypsin (TLCK-treated, Sigma-Aldrich) in reaction buffer (2 nM final). Inhibitor was added in 10 µL aliquots, as above. For comparative studies using chymotrypsin (2 µg/mL) and Der p 1 (20 µg/mL), the above conditions were modified to permit the inclusion of 10 µL DTT (1 mM final) and in these experiments AEBSF (400 µM) or ADZ 50,000 (40 µM) were used as inhibitors.

### 4.12. Analysis of Inhibitor Kinetics

Inhibitor kinetics were analysed by the progress curve method essentially as described [57]. For irreversible inhibitors, data were fitted by computational non-linear regression and the apparent inactivation rate constant ($k_{obs}$) was calculated from the Equation (1):

(1) $${\left[P\right]}_{t}=\frac{V_{z}}{k_{obs}}\left(1-e^{-k_{obs}\cdot t}\right)$$

where ${\left[P\right]}_{t}$ is the product concentration at time *t.*

$V_{z}$ is the velocity of the uninhibited reaction.

$k_{obs}$ is a pseudo first order rate constant.

For reversible inhibitors, IC_50_ values were calculated using conventional procedures.

### 4.13. Expression of Claudin 1.1

RNA was extracted from confluent cultures of Calu-3 cells grown as previously described [12,21,24,58] and cDNA synthesised according to manufacturer protocol (Qiagen, Manchester, UK). cDNA corresponding to the first extracellular domain of claudin 1, henceforth designated CLD1.1, was amplified using 5′-TAC ATA TGC AGT GGA GGA TTT ACT CCT ATG CCG GCG A-3′ as forward primer and 5′-ACG GAT CCT TAA CGG GTT GCT TGC AAT GTG CTG CTC AGA-3’ as reverse primer. PCR products were sub-cloned into pET 22b+ and transformed into *E. coli* expression strain BL21DE3 plySs. After induction by 1 mM IPTG this yielded a 6 kDa peptide on SDS-Tricine PAGE consistent with that predicted for CLD1.1 and which was absent in negative controls lacking IPTG. The CLD1.1 band was subjected to in-gel digestion by trypsin and the resultant fragments analysed by MALDI-TOF in positive reflectron mode. The resulting mass spectrum revealed two major fragments (*m/z* 620.2 and *m/z* 1764.6) which correspond to the sequences ^1^Met-Gln-Trp-Arg^4^ and ^39^Val-Phe-Asp-Ser-Leu-Leu-Asn-Leu-Ser-Ser-Thr-Leu-Gln-Ala-Thr-Arg^54^ confirming identity with a residue coverage of 37%.

To study its proteolysis by Der p 1, recombinant CLD1.1 was separated by SDS-Tricine PAGE and the band containing the peptide excised from the gel. The gel slices were successively washed with 10 mM ammonium bicarbonate buffer (pH 8.9), 50% MeCN and 100% MeCN and then freeze-dried. Reactions were performed by exposing the dried gel slices to Der p 1 in PBS containing 1 mM DTT and incubating at room temperature. Inhibitor studies were performed by pre-treating Der p 1 (4 µM) with either ADZ 50,000 (400 µM) or AEBSF (4 mM) for 10 min prior to addition to CLD1.1 with a 1:10 *v/v* dilution. Negative controls comprised Der p 1 or CLD1.1 incubated alone in reaction buffer. After treatment with Der p 1, 10 µL of supernatant from each reaction mixture was analysed by LC-MS. Capillary liquid chromatography was performed using a 5 µm reversed-phase column (BioBasic-18, 100 × 0.18 mm; ThermoElectron, Hemel Hempstead, Hertfordshire, UK) at 2 µL/min using a Surveyor MS pump coupled to the electrospray source of an ion trap mass analyser (LCQ Deca XP Plus, ThermoElectron, UK). Samples were eluted by gradient chromatography (0.1–30% B in A over 35 min, 30–50% B in A over 10 min followed by 50–80% B in A over 5 min, where A was aqueous 0.1% *v/v* formic acid and B was 0.1% *v/v* formic acid in MeCN. Mass spectra were acquired in full scan mode (*m/z* 300–1800) and the three most abundant ions subjected to MS/MS analysis in data-dependent acquisition mode with dynamic exclusion. Acquired MS/MS spectral data were searched against a custom database created for this experiment using BioworksBrowser and the TurboSEQUEST algorithm. Search parameters were declared as free cysteine and undefined enzyme. The molecular mass range was set to 600–3500 Da and filters applied as follows (Δcn: ≥0.1; Xc (± 1, 2, 3) 1.5, 2.0, 2.5; protein probability: ≤1 × 10^−3^. Fragments were evaluated according to their scores to verify the characterisation.

### 4.14. Cell Culture

Calu-3 cells (American Type Culture Collection) were cultured as previously described by us and is a favoured model system because they are of human origin, retain effective tight junctions (TJs) and form polarised monolayers which develop substantial transepithelial resistance [24,58]. These cells are well characterised as responding to HDM allergens through various mechanisms present in primary cultures of human airway epithelial cells [12,20,21,58].

For experiments involving measurements of permeability and transepithelial electrical resistance (TER), calu-3 cells were grown to confluence on 6.5 mm diameter, 0.1 µm pore polycarbonate Transwell filters (Corning^®^ Costar^®^) as described [14]. Treatments in serum-free minimum essential medium with Earle’s salts (EMEM) were applied to the apical compartment of each well and the plates incubated at 37 °C in a humidified 5% CO_2_/air atmosphere. TER was measured using an EVOM epithelial volt/ohm meter (World Precision Instruments, Sarasota, FL, USA) and medium harvested from both compartments at the end of the experimental treatment. 

A549 cells were cultured in 12-well plates (Corning^®^ Costar^®^) using methods described elsewhere [58]. At confluence, cells were washed with serum-free EMEM and experimental treatments applied for 8 h at 37 °C in a final volume of 1 mL. Treatment medium was harvested and replaced by 1 mL serum-free medium and incubation continued for 16 h. The medium was harvested, centrifuged (10,000× *g*, 30 s) to sediment non-adherent cells and the supernatants stored at −20 °C. Whole cell protein extracts were prepared by the addition of 1 mL boiling 2% SDS *w/v* in 312 mM Tris, pH 6.8). Protein concentration was measured using the Biuret method.

### 4.15. Studies Performed In Vivo

These studies were compliant with requirements of the Animals Scientific Procedures Act (UK) in an AAALAC-accredited facility and were subject to prior ethical review. Acute tolerability investigations performed with the test substances prior to conduct of these studies did not reveal any obvious adverse events over a 24 h period following dosing.

### 4.16. Allergic Responses in Rats

Animals (Brown Norway, 250–350 g, Charles River, Ormiston, UK) were housed under isolation and randomly assigned to treatment groups. Rats were sensitised to HDM allergen mixture, with aluminium hydroxide adjuvant, or vehicle on days 0, 7, and 14 by intraperitoneal (i.p.) injection (0.5 mL). On day 17 the animals were briefly anaesthetised with isoflurane in oxygen and sensitisation treatments administered as intratracheal aerosols (delivery volume to airways 100 µL) using a Penn-Century IA-1C/FMJ-250 dosing device. 

For measurement of acute allergic bronchoconstriction and airways hyperreactivity, animals were anaesthetised on day 21 with pentobarbitone (100 mg/kg i.p.) and ventilated via a tracheal cannula (approximately 7 mL/kg, 1 Hz) with a 50:50 *v/v* mixture of oxygen in air. The anaesthetised, ventilated animals were then paralysed with norcuron (4 mg/kg i.m., MSD, Hoddesdon, Hertfordshire, UK). Ventilation was monitored using a flow transducer (Fleisch, type 0000) in-line with the respiratory pump. Coincident pressure changes within the thorax were monitored directly using an intrathoracic cannula, enabling display of the pressure differential between trachea and thorax. Airways resistance (R_L_) and dynamic compliance (C_dyn_) were calculated for each respiratory cycle on a digital electronic respiratory analyser (PMS, Mumed Ltd., London, UK). Blood pressure and heart rate were also routinely recorded. Animals were challenged by vehicle of HDM allergen mixture (with or without ADI compounds) using a Penn-Century aerosoliser inserted into the trachea. Dose delivery volume to the airways was 100 µL. Acute bronchoconstrictor responses arising from the challenge were measured as the change in R_L_ from baseline. One hour after challenge the responses to a dose of acetylcholine (100 µg/kg, 1 mL/kg) and then to a dose of adenosine (10 mg/kg, 1 mL/kg) were recorded. At the end of the study, the animals were euthanised by pentobarbitone overdose. As a further control, a group of sensitised animals was challenged by i.t. aerosol on day 21 and airways hyperreactivity measurements made 24 h later as described above.

For studies of allergen-induced leukocyte accumulation, animals were briefly anaesthetised (isoflurane in oxygen) on day 21 after the beginning of the sensitisation procedure and vehicle, HDM allergen mixture or HDM allergen mixture with ADI compound delivered from a Penn-Century aerosoliser as previously described. Animals were then allowed to recover from anaesthetic to enable assessment of cell recruitment to the lungs to be made 48 h after challenge. At this point animals were killed with pentobarbitone (250 mg/kg i.p.) and the lungs lavaged via a tracheal cannula using 3 × 4 mL aliquots of Hanks’ balanced salt solution (HBSS) containing 10 mM EDTA and 25 mM HEPES. The recovered cells from each animal were pooled and the total volume recovered adjusted to 12 mL using HBSS. Total cells were counted (ADVIA®, Bayer Healthcare, Diagnostic Division, Reading, Berkshire, UK) and smears made by diluting recovered fluid (to approximately 106 cells/mL) and pipetting an aliquot (100 mL) into a centrifuge (Cytospin, Shandon, UK). Smears were air-dried, fixed for 10 s using methanol, and stained with buffered eosin (10 s) and methylene blue/Azur (5 s) (Speedy-Diff, Clin-Tech Ltd., Guildford, Surrey, UK) to differentiate eosinophils, neutrophils, macrophages/monocytes, and lymphocytes. A total of 500 cells in each sample were counted by light microscopy at ×1000 using an oil immersion objective.

In both studies, a terminal blood sample was taken from each animal and serum prepared for the measurement of immunoglobulins.

### 4.17. Allergic Sensitization Studies in Mice

Mice (female Balb/c or C57BL/6 depending on study, 20 ± 2 g, Charles River/BioLASCO) were maintained in an isolator facility using individually ventilated cages (Allentown IVC Racks, 36 Mini Isolator System, Allentown, NJ, USA) which had been autoclaved before use. The environmental regime was 22–24 °C/60–80% relative humidity on a 12 h light/dark cycle. There was ad libitum access to reverse osmosis-purified water and food (MF-18 laboratory rodent diet). Where the investigation required both pre- and post-study serum samples, the pre-study samples were taken from the retro-orbital sinus on acclimatisation in the isolator. Animals were randomly assigned to groups and sensitised to a natural mixture of HDM allergens, Der p 1 alone, Der p 2 alone, or treated with vehicle on day 0, 7, and 14. The route of administration, i.p. or intranasal, was according to predetermined study protocol. Animals were challenged under anaesthetic cover by i.t. aerosol on day 21 using a Penn-Century IA-1C/FMJ-250 aerosoliser (20 µL/mouse). On day 22 the animals were anaesthetised with propofol (AstraZeneca, 10 mg/mL, 50 µL/mouse, i.v.) and terminal blood samples taken from the retro-orbital sinus. Then BAL was performed using 2 × 0.25 mL or 3 × 0.5 mL aliquots of PBS according to study design. The returns were combined, fluid volumes measured, and cells enumerated (Sysmex XT-1800iV, Sysmex, Kobe, Japan). A similar approach was used in studies involving ovalbumin (OVA) challenge in animals sensitised i.p. to OVA with Al(OH)_3_ as adjuvant.

### 4.18. ELISA Measurements

Cytokines were assayed using kits and according to manufacturer protocol (Biosource Europe, Nivelles, Belgium). Der p 1 and Der p 2 were quantified by ELISA using reagents obtained from Indoor Biotechnologies, UK. 

Total IgE was measured by sandwich ELISA in Corning^®^ Costar^®^ high binding 96-well microplates. For rat serum, plates were coated overnight with mouse anti-rat IgE (clone B41-1, BD Biosciences Europe, Wokingham, Berkshire, UK) in carbonate-bicarbonate buffer and blocked using DPBS with 0.05% *v/v* Tween^®^ 20 (DPBST) containing 10% *v/v* foetal bovine serum. Rat serum samples or IgE standards (BD Biosciences) were diluted in Can Get Signal^®^ Solution 1 (2B Scientific, Upper Heyford, Oxfordshire, UK) and incubated on the plate for 15 h at 4 °C. After washing, the secondary antibody (biotin-conjugated mouse anti-rat, clone B41-3, BD Biosciences) and streptavidin-horseradish peroxidase were added in Can Get Signal^®^ Solution 2 and the plate incubated at room temperature for 3.5 h. After washing, substrate solution (3,3′,5,5′-tetramethylbenzidine dihydrochloride in phosphate-citrate perborate buffer) was added to each well and the reaction terminated by addition of 2 M sulphuric acid after incubation in the dark for 5–15 min. Plates were read at 450 nm using an automated plate analyser and data quantified by interpolation from standard curves. A similar procedure was used for the measurement of mouse IgE using an OptEIA™ set (BD Biosciences).

Allergen-specific immunoglobulin assays were performed by indirect ELISA. Plates were coated overnight at 4 °C with Der p 1 or mixed HDM allergens in carbonate-bicarbonate buffer and blocked according to our standard procedures. Samples of serum were then added and incubated as described before addition of the appropriate rat (RG7/1.30 or B41-3, BD Biosciences) or mouse (BD OptEIA or MCA336P, AbD Serotec, Kidlington, UK) secondary antibodies diluted in Can Get Signal^®^ Solution 2. Assays were developed as described above and data reported as absorbance at 450 nm.

### 4.19. BAL Analysis by Flow Cytometry

Polychromatic flow cytometry of BAL fluid samples was performed on a BD FACSAria™ instrument (Becton Dickinson Biosciences, Franklin Lakes, NJ, USA) with data acquisition and analysis performed using FACSDiva™ software. Unless stated otherwise, antibodies for these studies were obtained from BD Pharmingen and comprised MHC class II-FITC conjugate (antibody 2G9), CD11c-allophycocyanin conjugate (antibody HL-3), CD11c-phycoerythrin/Cy7 conjugate, CD3-phycoerythrin/Cy5 conjugate (antibody 145-2C11), B220 (CD45R)-phycoerythrin/Cy5 conjugate (antibody RA3-6B2), CCR3-phycoerythrin (antibody 83101, R&D Systems, Abingdon, Oxfordshire, UK). FcγR blocking agent was antibody 2.4G2. Dendritic cells were enumerated as non-autofluorescent CD3/B220^−^, MHC II^+^, CD11c^+^. Eosinophils were SSC^high^, CCR3^+^, moderate CD11c^+^, low-absent MHC II, B220/CD3^−^.

### 4.20. Data Presentation and Statistical Analyses

Data are presented as mean values ± s.e. from the indicated number of observations. Significance was determined using one-way analysis of variance with *post hoc* testing using the Student–Newman–Keuls procedure in SigmaPlot vs. 12.0 unless stated otherwise. A probability value of *p* < 0.05 was considered statistically significant.

## Figures and Tables

**Table 1:** Measured Km values for ADZ 50,059 against Der p 1 and a panel of cysteine and serine peptidases.

Enzyme	Km (µM)
Der p 1	15.4 ± 2.1
Der f 1	29.0 ± 2.6
Cathepsin B	40.9 ± 4.5
Cathepsin L	8.0 ± 1.3
Cathepsin S	12.3 ± 1.8
Trypsin	46.6 ± 2.9
Chymotrypsin	132.1 ± 22.7

Data are mean ± s.e. mean from 4 determinations.

**Table 2:** Measured activity of ADZ 50,000 against Der p 1, Der f 1, and a panel of off-target cysteine and serine peptidases.

Enzyme	*K_obs_*/[*I*] (M^−1^ s^−1^)
Der p 1	2 × 10^8^
Der f 1	1.9 × 10^8^
Cathepsin B	3.7 × 10^7^
Cathepsin S	2.1 × 10^7^
Cathepsin L	1.7 × 10^6^
Cathepsin H	1.5 × 10^6^
Cathepsin K	1.6 × 10^4^
Thrombin	inactive at ≤50 µM
Trypsin	inactive at ≤50 µM
Chymotrypsin	inactive at ≤50 µM

**Table 3:** Cleavage sites for Der p 1 identified in CLD1.1. Peptides identified by LC-MS-MS were used to identify positional cleavages indicated by single letter amino acid notation in the first column. Implied subsite specificity of Der p 1 is indicated for positions S_4_–S_4′_. Similarity with potential cleavage sites in the first extracellular domain of occludin are highlighted in yellow. Grey and blue shading denote non-prime and prime subsite interactions of Der p 1 relative to the cleavage site (Schecter and Berger nomenclature).

TJ Loop and Identified Cleavage	S_4_	S_3_	S_2_	S_1_	S_1′_	S_2′_	S_3′_	S_4′_
**CLD1.1**								
SLLN.LSST	Ser	Leu	Leu	Asn	Leu	Ser	Ser	Thr
FDSL.LNLS	Phe	Asp	Ser	Leu	Leu	Asn	Leu	Ser
LSST.LQAT	Leu	Ser	Ser	Thr	Leu	Gln	Ala	Thr
IVTA.QAMY	Ile	Val	Thr	Ala	Gln	Ala	Met	Tyr
IQCK.VFDS	Ile	Gln	Cys	Lys	Val	Phe	Asp	Ser
RIYS.YAGD	Arg	Ile	Tyr	Ser	Tyr	Ala	Gly	Asp
CVSQ.STGQ	Cys	Val	Ser	Gln	Ser	Thr	Gly	Gln
VTAQ.AMYE	Val	Thr	Ala	Gln	Ala	Met	Tyr	Glu
**Occludin 1.1**								
GTSL.LGGS	Gly	Thr	Ser	Leu	Leu	Gly	Gly	Ser

**Table 4:** Similarity matrix for first extracellular domains of human claudin (CL) family adhesion proteins created using Clustal Omega. Grey shading indicates claudins expressed in human bronchi and bronchioles. Note that: (i) some evidence indicates that claudin-7 may be an anion channel-forming rather than a sealing type claudin; (ii) the similarity data for claudin 10 are based on the canonical claudin 10b sequence; (iii) the data for claudin 18 are based on the canonical sequence of the A1 isoform (UniProt: P56856).

CL	1	2	3	4	5	6	7	8	9	10	11	12	14	15	16	17	18	19	20	21	22	23	25
**1**	100	43	61	62	57	60	70	51	60	37	29	24	49	38	26	42	44	64	38	34	34	25	30
**2**	43	100	45	45	42	49	47	40	49	35	29	22	58	30	26	36	42	55	40	24	24	19	26
**3**	61	45	100	94	67	80	59	71	80	44	32	25	61	42	31	63	42	57	45	40	40	24	31
**4**	62	45	94	100	74	85	55	68	83	42	35	28	62	40	34	58	46	53	43	38	38	25	34
**5**	57	42	67	74	100	72	60	57	70	40	37	24	53	36	34	43	40	49	45	36	36	25	30
**6**	60	49	80	85	72	100	53	66	98	40	35	26	59	32	32	53	42	57	43	40	40	23	34
**7**	70	47	59	55	60	53	100	49	53	42	33	26	49	38	32	43	42	60	45	36	36	25	28
**8**	51	40	71	68	57	66	49	100	66	33	31	28	49	32	34	70	37	45	32	40	40	25	32
**9**	60	49	80	83	70	98	53	66	100	40	35	26	58	32	32	53	40	57	43	40	40	23	34
**10**	37	35	44	42	40	40	42	33	40	100	35	18	37	64	30	31	29	42	37	30	29	13	28
**11**	29	29	32	35	37	35	33	31	35	35	100	17	37	24	27	27	33	32	38	27	26	19	27
**12**	24	22	25	28	24	26	26	28	26	18	17	100	22	23	27	20	27	24	22	21	21	24	19
**14**	49	58	61	62	53	59	49	49	58	37	37	22	100	34	34	45	38	47	47	32	32	23	30
**15**	38	30	42	40	36	32	38	32	32	64	24	23	34	100	27	34	28	38	32	22	22	16	20
**16**	26	26	31	34	34	32	32	34	32	30	27	27	34	27	100	30	25	28	26	32	31	19	26
**17**	42	36	63	58	43	53	43	70	53	31	27	20	45	34	30	100	31	47	36	36	36	25	32
**18**	44	42	42	46	40	42	42	37	40	29	33	27	38	28	25	31	100	38	35	28	27	29	28
**19**	64	55	57	53	49	57	60	45	57	42	32	24	47	38	28	47	38	100	43	32	32	27	28
**20**	38	40	45	43	45	43	45	32	43	37	38	22	47	32	26	36	35	43	100	24	24	21	28
**21**	34	24	40	38	36	40	36	40	40	30	27	21	32	22	32	36	28	32	24	100	100	24	62
**22**	34	24	40	38	36	40	36	40	40	29	26	21	32	22	31	36	27	32	24	100	100	25	62
**23**	25	19	24	25	25	23	25	25	23	13	19	24	23	16	19	25	29	27	21	24	25	100	18
**25**	30	26	31	34	30	34	28	32	34	28	27	19	30	20	26	32	28	28	28	62	62	18	100

## Abbreviations

Abz	aminobenzoyl
ACh	acetylcholine
ADI	allergen delivery inhibitor
Ado	adenosine
ADZ 50,059	(3*S*,6*S*,9*S*,12*S*,15*S*,18*S*)-1-(2-aminophenyl)-9-butyl-18-carbamoyl-15-(4-hydroxy-3-nitrobenzyl)-12-(hydroxymethyl)-3-isopropyl-6-methyl-1,4,7,10,13,16-hexaoxo-2,5,8,11,14,17-hexaazaicosan-20-oic acid
AEBSF	4-(2-aminoethyl)benzenesulphonyl fluoride
AMC	7-amino-4-methylcoumarin
BAL	bronchoalveolar lavage
Bz	benzoyl
CH_2_Ph	benzyl
CLD1.1	extracellular domain 1 of claudin-1
DCM	dichloromethane
Der f	*Dermatophagoides farinae*
Der p	*Dermatophagoides pteronyssinus*
DMF	dimethylformamide
DMSO	dimethyl sulphoxide
DNP	2,4-dinitrophenol
DTT	dithiothreitol
E-64	*trans*-epoxysucciny-l-leucyl-amido (4-guanidino) butane
EDTA	ethylenediaminetetraacetic acid
EMEM	minimum essential medium with Earle’s salts
GM-CSF	granulocyte macrophage-colony stimulating factor
HBSS	Hanks’ balanced salt solution
HDM	house dust mite
HEPES	4-(2-hydroxyethyl)-1-piperazine ethanesulphonic acid
HPLC	high performance liquid chromatography
HTS	high-throughput screening
i.p.	intraperitoneal
iPr	isopropyl
IL	interleukin
IPTG	isopropyl β-d-1-thiogalactopyranoside
i.t.	intratracheal
LC-MS	liquid chromatography-mass spectrometry
MALDI-TOF	matrix-assisted laser desorption ionisation time-of-flight mass spectrometry
MeCN	acetonitrile
MES	2-morpholinoethanesulphonic acid
NaAc-AcH	sodium acetate-acetic acid
NMR	nuclear magnetic resonance
OVA	ovalbumin
PAGE	polyacrylamide gel electrophoresis
PAR	protease-activated receptor
PBS	phosphate-buffered saline
SDS	sodium dodecyl sulphate
TER	transepithelial electrical resistance
THF	tetrahydrofuran
TJ	tight junction
TLCK	Nα-*p*-tosyl-l-lysine choromethyl ketone
TPCK	N-*p*-tosyl-l-phenylalanine chloromethyl ketone
